# Engaging learners with games–Insights from functional near-infrared spectroscopy

**DOI:** 10.1371/journal.pone.0286450

**Published:** 2023-06-06

**Authors:** Melina De Nicolò, Thomas Kanatschnig, Manuel Hons, Guilherme Wood, Kristian Kiili, Korbinian Moeller, Simon Greipl, Manuel Ninaus, Silvia Erika Kober

**Affiliations:** 1 Institute of Psychology, University of Graz, Graz, Austria; 2 BioTechMed-Graz, Graz, Austria; 3 Faculty of Education and Culture, Tampere University, Tampere, Finland; 4 Centre for Mathematical Cognition, School of Science, Loughborough University, Loughborough, United Kingdom; 5 Leibniz-Institut für Wissensmedien, Tübingen, Germany; 6 LEAD Graduate School & Research Network, University of Tübingen, Tübingen, Germany; 7 Department of Media and Communication, Ludwig Maximilian University of Munich, Munich, Germany; Kyoto University Graduate School of Informatics: Kyoto Daigaku Daigakuin Johogaku Kenkyuka, JAPAN

## Abstract

The use of game elements in learning tasks is thought to facilitate emotional and behavioral responses as well as learner engagement. So far, however, little is known about the underlying neural mechanisms of game-based learning. In the current study, we added game elements to a number line estimation task assessing fraction understanding and compared brain activation patterns to a non-game-based task version. Forty-one participants performed both task versions in counterbalanced order while frontal brain activation patterns were assessed using near-infrared spectroscopy (within-subject, cross-sectional study design). Additionally, heart rate, subjective user experience, and task performance were recorded. Task performance, mood, flow experience, as well as heart rate did not differ between task versions. However, the game-based task-version was rated as more attractive, stimulating and novel compared to the non-game-based task version. Additionally, completing the game-based task version was associated with stronger activation in frontal brain areas generally involved in emotional and reward processing as well as attentional processes. These results provide new neurofunctional evidence substantiating that game elements in learning tasks seem to facilitate learning through emotional and cognitive engagement.

## Introduction

The use of game elements in learning tasks has become increasingly popular in recent years [1]. Including game elements should lead to increased interest of learners and adherence to instruction compared to non-game based task versions [2–4]. Additionally, game-based learning environments were found to either increase performance in various contexts or at least yield a performance comparable to those observed in traditional non-game-based environments [2, 5].

One focus of research in the context of game-based learning has been on mathematics education and the field of number knowledge, especially on facilitating fraction understanding [4, 6, 7]. Number line estimation task [8] is a frequently used learning mechanic in game-based learning studies focusing on fractions [6]. In number line estimation task learners are to estimate, for instance, the position of a fraction on a number line ranging from 0 to 1 [9]. A game-based implementation of the number line estimation task typically includes game elements such as a narrative and virtual incentives, while a non-game-based task version does not include such game elements. Studies employing game-based number line estimation tasks for fraction learning reported significant increases in performance reflecting improvements in participants’ fraction understanding [10, 11].

While the number of studies addressing behavioral effects of game-based learning is increasing, the neural mechanisms and underpinnings of the respective learning processes in game-based learning were investigated only rarely. Nevertheless, first neuroimaging studies evaluating the neural correlates of game-based learning indicated that game-based learning seems to facilitate the learning process through processes of reward, attention, and emotional engagement [12, 13]. In particular, the studies by Greipl et al. [13] and Kober et al. [12] both compared neural correlates of a game-based version as well as a non-game-based version of a number line estimation task. Kober et al. [12] used near-infrared spectroscopy (NIRS) to measure changes in the hemodynamic response over frontal brain areas, while Greipl et al. [13] used functional magnetic resonance imaging (fMRI). Both studies found that the game-based task version leads to more pronounced activation in brain areas associated with reward processing (e.g., the orbitofrontal cortex) than a non-game-based task. This is in line with a number of other studies indicating that game elements may generally activate the reward system in the brain [14–20]. In the fMRI study by Greipl et al. [13], activation in brain areas associated with emotional processing, such as the amygdala and anterior insula, was stronger in the game-based compared to the non-game-based condition. The NIRS study by Kober et al. further indicated stronger activation in frontal brain areas associated with attentional processes [12]. Note that in the fMRI study by Greipl et al. [13] feedback episodes were analyzed while in the NIRS study by Kober et al. [12] the whole task including number line estimation as well as feedback episodes was analyzed. In sum, these prior neuroimaging studies indicate on a neurofunctional level that including game elements in learning tasks seems to facilitate learning through attentional processes, reward processing, and emotional engagement [12, 13].

In the present study, our goal was to replicate these previous findings by investigating the hemodynamic response in frontal brain areas using NIRS while participants perform a game-based and a non-game-based version of a number line estimation task. We used the same task as Greipl et al. [13] used in their fMRI study. NIRS is a portable, flexible and easy-to-use method to measure hemodynamic changes at the cortex level. In the past decade, the use of NIRS as a method in gaming settings became more frequent [21, 22] as it provides several benefits: i) it is relatively insensitive to participants’ movements, ii) it can be combined with a variety of other signal acquisition methods or tools, iii) it offers a quite comfortable and authentic work [23, 24], iv) and gaming environments can be maintained during studies so that the gaming situation can proceed undisturbed during scientific measurements [25]. This is also relevant in the context of game-based learning, as authentic learning environments play an important role in studies focusing on a better understanding of the mechanisms behind educational activities [26]. Replicating the results of previous fMRI measurements with NIRS would therefore increment ecological validity of brain activation monitoring during intervention studies with game-based learning in the future.

Based on previous findings, we expected stronger frontal brain activation in the game-based compared to the non-game-based condition due to a heightened attentional focus as well as emotion and reward processing in the game-based task version. These factors might also be facilitated by the fact that the game-based task was designed in an intrinsically integrated way by combining content knowledge (fractions), content specific instructional knowledge (number line estimation) and game mechanics/elements in a meaningful way [27]. Such intrinsic integration is supposed to increase intrinsic motivation and the effectiveness of learning [28]. As in the studies by Greipl et al. [13] and Kober et al. [12], expected differences in brain activation between task versions should be related to the intrinsically integrated game elements (for more details see Methods), as math content, learning mechanic and task complexity were held constant across the game-based and the non-game-based version. According to previous research the game elements in the game-based task should be rewarding and emotionally engaging [29–31], thereby leading to stronger frontal brain activation compared to the non-game-based task.

In line with previous studies, we also assessed user experience (in terms of mood, flow, and user experience) and compared it between task versions. Game-based tasks are often experienced as more interesting, attractive, novel, and stimulating [12, 13, 32], while non-game-based tasks might be rated as more efficient [12, 13]. Since it is expected that a game-based task should lead to stronger emotional processing than a non-game-based task, affective states such as mood might also differ between task versions [12].

As a complement to previous studies [12, 13, 32], we also assessed physiological responses, i.e., changes in heart rate, while participants performed both tasks. Differences in emotional engagement between task versions might also lead to differences in heart rate [33, 34].

When comparing game-based and non-game-based task versions, the question always arises whether performance measures differ between task versions. As mentioned before, previous studies reported heterogenous results. Adding game elements to learning tasks either increased performance or led to a comparable performance than traditional non-game-based task versions [2, 5]. Previous studies using a game-based and non-game-based number line estimation task comparable to the present study found a comparable task performance in healthy young adults [12, 13, 32].

## Methods

### Participants

Forty-eight participants (25 women and 23 men) between 18 and 30 years of age participated in the study. Due to technical issues and therefore incomplete data, five participants had to be excluded from the sample. Furthermore, two participants could not complete the study due to headaches and nausea during the measurement period. Therefore, the final sample included 41 participants (20 women and 21 men), who were, on average, 22.95 years old (*SD* = 3.04). All participants had normal or corrected-to-normal vision. Exclusion criteria included any severe illnesses, neurological or psychiatric diagnoses, cardiovascular diseases, or use of medication affecting the central nervous system or participants’ attention and vigilance. In addition, participants had to be eligible for NIRS measurements, meaning that they should not have, e.g., any wounds or inflammation around their heads and on their scalp or uncontrollable muscle spasms. We also assessed the video game habits of the participants using self-assessment. Regarding their video game habits, 32% of participants reported that they had not played video games in the 12 months prior to their participation in the study. Most participants (49%) rarely or sometimes played video games, while 17% reported that they played video games frequently or almost daily. Participants were recruited through a university-wide newsletter, social media, and personal recruitment. As compensation for their participation, psychology students at the University of Graz received course credit (note that not all participants were psychology students). No financial compensation was offered to participants. All participants gave written informed consent prior to the study, which was approved by the local ethics committee of the University of Graz, Austria (reference number GZ. 39/35/63 ex 2020/21) and is in accordance with the ethical standards of the Declaration of Helsinki. Two of the authors (MDN and SEK) had access to information that could identify individual participants during and after data collection.

### Game-based vs. non-game-based task version

All participants performed both a game-based and a non-game-based version of a number line estimation task. Half of the participants started with the game-based version, and the other half with the non-game-based version.

The game-based task version used in this study was based on the *Number Trace* game [10], which uses (fraction) number line estimation tasks as its core learning (game) mechanic. The current version of the game was specifically created for fMRI and NIRS research. Only a limited number of game elements were included in this version to ensure that any differences in brain activation patterns between the game-based and non-game-based task versions are specifically related to these game elements and that the game elements do not cause cognitive overload. That is, only game elements of a controllable avatar (a dog), visually appealing game world (forest) and avatar-based emotional feedback (facial expressions of the dog) were included and integrated to the core learning mechanic (number line estimation). This same version has previously been used in the fMRI study by Greipl et al. [13]. A more in-depth description of the game can be found in their paper.

**Fig 1:**
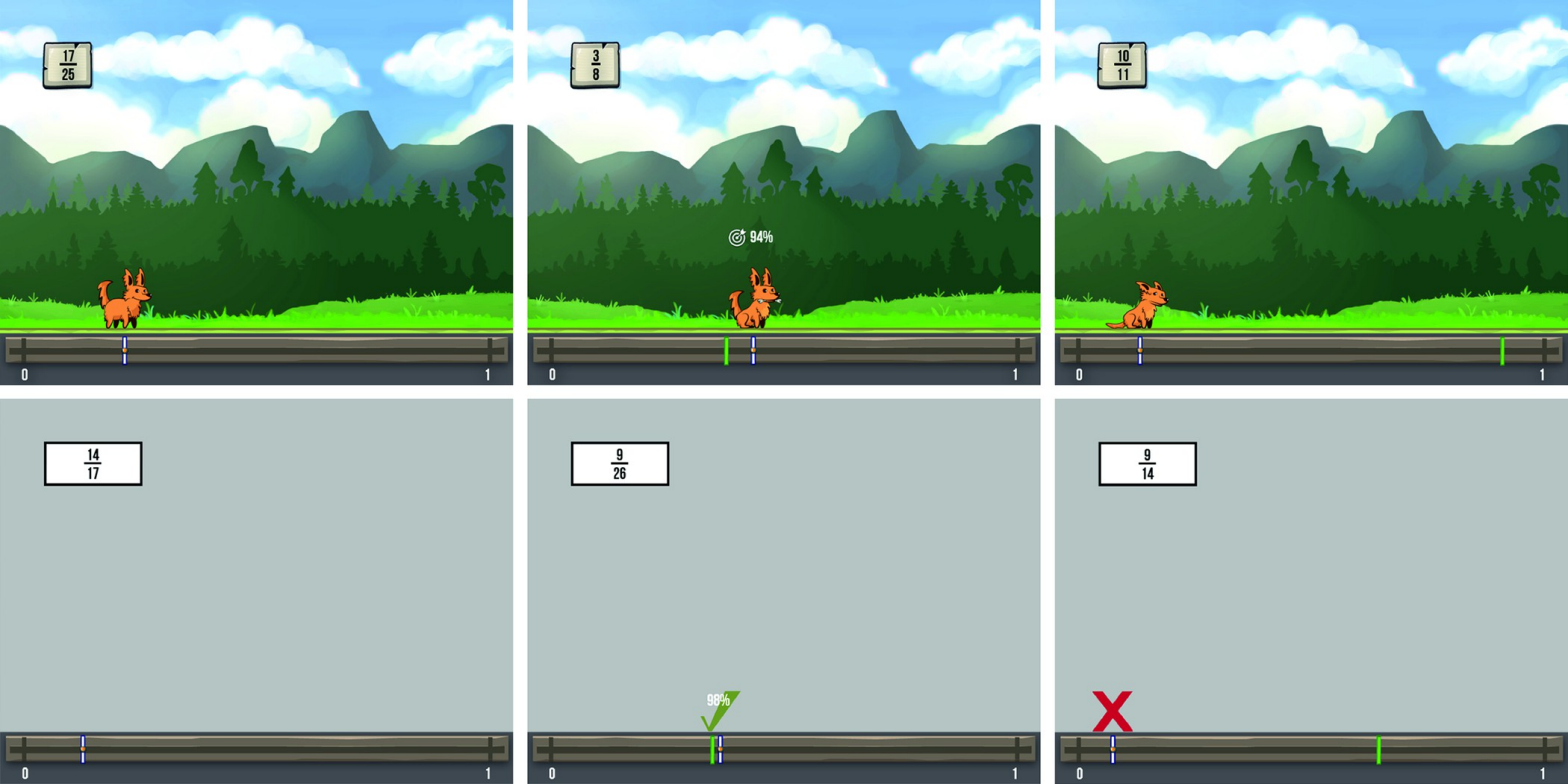
Example scenes of task versions. Exemplary screenshots of the game-based version (upper row) and the non-game-based version (lower row) of the number line estimation task. Screenshots in the left column show the two versions of the task during the estimation process. The screenshots in the middle column and those in the right column show positive feedback after correct estimation as well as negative feedback after incorrect estimation, respectively.

In general, the goal of the player in the current version of Number Trace was to correctly estimate the location of fractions on a number line ranging from 0 to 1. The fraction that was to be estimated was displayed in a box in the upper left corner of the screen (Fig 1). To enter their estimations, players had to navigate an avatar in the form of an orange dog along the number line using the arrow keys on a standard QWERTZ computer keyboard. The chosen position was then confirmed by pressing the space bar, which caused the dog to start digging. If the position was at least 90% accurate (i.e., located within +/- 10% of the correct location of the fraction), the answer was deemed correct, and the dog was rewarded with a bone and smiled. When the chosen position was false (i.e., located outside of +/- 10% of the correct position), the dog did not receive any bones and started to cry. In both cases, the accurate position of the fraction was indicated by a green bar. When the estimation was at least 90% accurate, estimation accuracy was additionally displayed on the screen (Fig 1).

This version of Number Trace also included a control condition during which players had to simply choose a position on the number line instead of estimating a fraction with a bone indicating the target position, and instead of a fraction, letter pairs were displayed in the box in the upper left corner. As this condition was mainly used to investigate differences between the processing of fractions and non-numerical information, which was not the objective of this study, only the fraction condition of the game was analyzed in this study.

In total, participants had to complete one game-based level consisting of 24 item blocks with four items each. Twelve blocks included only fraction estimation items, while the other twelves included only letter pair items. After each block, there was a 23 second break in which only the background picture of the game was displayed on the screen. Players had ten seconds per item to estimate the correct position and confirm their answer. If they did not choose a position on the number line within this time frame, the software automatically logged in the position the avatar was located at. Depending on the time the player took to answer, the duration of the game level was between 18 and 25 minutes (i.e., all 24 item blocks).

As described by Greipl et al. [13], all fraction items used in the game-based task version Number Trace and the corresponding non-game-based version contained numerators and denominators ranging from 2 to 29. At the start of any level of the game or non-game-based version all of the displayed items were randomly drawn from a pool of possible items by the game engine. Thus, items were presented to each participant in a randomized order and also differed between task blocks as well as different levels of the game and non-game-based version. The overall item and task block difficulty was comparable for all levels of both task versions.

The non-game-based task used in this study was identical to the game-based task regarding its composition, goal, and task difficulty. Again, users had to estimate fractions on a number line using the same controls as in the game-based version of the task. However, instead of controlling an avatar, users could only change the position of a white, vertical bar on the horizontal number line, without any other colorful and game-like elements. Analogous to the game-based task, answers with at least 90% accuracy were deemed correct and estimation accuracy was displayed on the screen accompanied by a green tick for these estimations. A red X was displayed in case the answer fell outside the range of estimations deemed correct. These two different feedback screens can be seen in Fig 1. In both cases, the correct position of the fraction was indicated by a green, vertical bar. In the letter pairs condition, the position users had to navigate to was also indicated by this green bar.

Again, in the non-game-based version of the task, users completed one level, which consisted of twelve blocks of four fraction estimation items and twelve blocks of four letter pairs items. After each block, there was a 23-second break, with the overall duration of the level also being between 18 and 25 minutes.

In both task versions, mean accuracy across participants’ estimation on all fraction estimation items as well as their hit rate were calculated for each condition. In this case, the hit rate reflected the percentage of correct estimations automatically determined by the software.

### NIRS recordings and analysis

**Fig 2:**
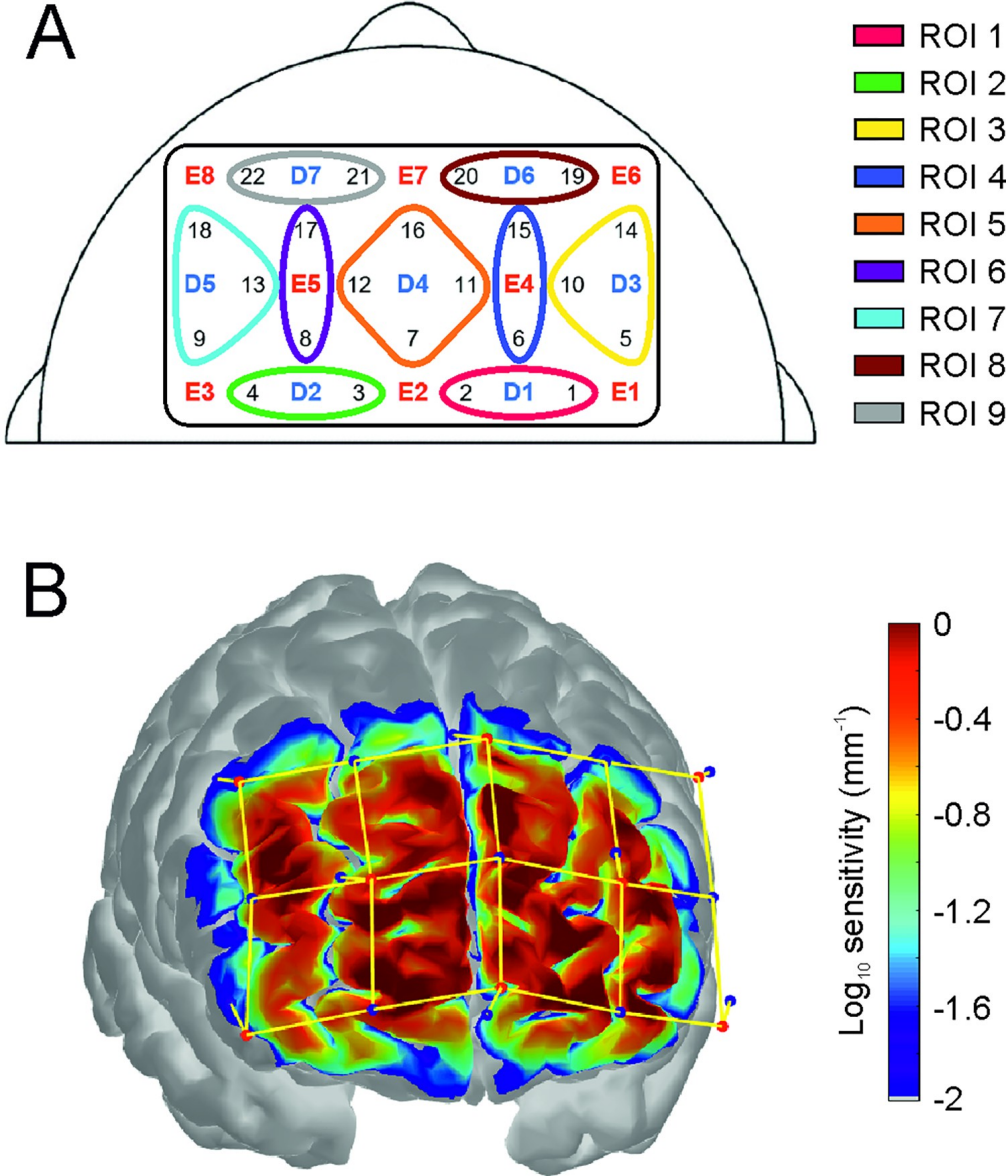
Position of NIRS optodes and sensitivity. (A) Placement of the 8 emitters [E], 7 detectors [D] and 22 NIRS channels (numbers written in black represent the respective NIRS channel number) on the forehead. Note that the 8 short-distance detectors, which were placed next to each emitter, are not shown in the figure. Positions of the 9 regions of interests (ROIs) are also color coded. (B) Sensitivity profile showing the coverage of the frontal cortex.

NIRS measures relative concentration changes in oxygenated (oxy-Hb) and deoxygenated (deoxy-Hb) hemoglobin. Activation of a certain brain area leads to an influx of oxygen-rich blood to this active area as well as to surrounding tissue, which is accompanied by an increase in oxy-Hb and a decrease in deoxy-Hb in this active brain area [35–38]. To measure participants’ hemodynamic activation in the frontal cortex, a 22-channel NIRSport2 system (NIRx Medizintechnik GmbH, Berlin, Germany) and the accompanying software Aurora fNIRS (version 1.4; NIRx Medizintechnik GmbH, Berlin, Germany) were used. The NIRS setup in this study consisted of eight emitter- and seven detector-optodes resulting in 22 long-distance NIRS channels. In addition, eight short-distance detectors with an emitter-detector distance of 8 mm were also included in this setup. Short-distance detectors were used to measure activation in the superficial extra-cerebral tissue, which was incorporated into NIRS data analysis to improve accuracy and reliability of results by reducing the influence of physiological artifacts [39, 40]. The approximate distance between emitters and detectors for the long-distance channels was 30 mm, and the NIRS system’s sampling rate was set to 10.17 Hz. The exact optode placements and resulting channels are depicted in Fig 2A.

For further data analysis, channels were merged into regions of interest (ROIs) following the procedure suggested by Kober et al. [12]: ROI 1: Superior frontal cortex right; ROI 2: Superior frontal cortex left; ROI 3: Middle frontal cortex right; ROI 4: Superior frontal cortex right; ROI5: Superior frontal cortex left and right; ROI 6: Superior frontal cortex left; ROI 7: Middle frontal cortex left; ROI 8: Orbitofrontal cortex right; ROI 9: Orbitofrontal cortex left. An overview of the ROIs can be found in Fig 2A.

The NIRS data was processed using the MATLAB-based package Homer2 (Ver. 2.8) [41]. To identify concentration changes in oxygenated (oxy-Hb) and deoxygenated hemoglobin (deoxy-Hb) during the fraction estimation periods, several processing steps were performed. First, the raw data was converted into optical density data using the function *hmrIntensity2OD*. Then, the function *enPruneChannels* (dRange -1e+04 1e+07, SNRthresh 2, SDrange 0.0 45.0, reset 0) was used to exclude channels with signals that deviated strongly from the other channels’ signals (e.g., channels with very low signal-to-noise ratio). Following that, motion artifacts were identified with a channel-wise approach (function *hmrMotionArtifactByChannel*; tMotion 0.5, tMask 1.0, STDEVthresh 10.0, AMPthresh 0.50).

A Spline motion correction was performed (function *hmrMotionCorrectSpline*; p = 0.99, turnon 1) followed by a Wavelet transformation (function *hmrMotionCorrectWavelet*; iqr = 1.5, turnon 1). The choice to use a combination of Spline interpolation and Wavelet transformation is based on a recommendation by 42 [42] who observed that this combination led to the best results in motion artifact correction and trial preservation. Different to 42 [42], a more lenient iqr value of 1.5 was used in the Wavelet transformation in order to avoid losing important information and excluding non-artifact data from the signal.

In a next step, motion artifacts were identified again (function *hmrMotionArtifact*; tMotion 0.5, tMask 1.0, STDEVthresh 50.0, AMPthresh 3.00). Then, the function *enStimRejection* was used to reject trials which started or ended in a time frame between 25.0 seconds before to 20.0 seconds after a motion artifact (tRange -25.0 20.0). After that, bandpass filters were applied to the data (*hmrBandpassFilt*; hpf 0.010, lpf 0.50) and the optical density data was converted into hemoglobin concentration changes (*hmrOD2Conc*; ppf 6.0 6.0). Finally, the data obtained from the short-distance detectors was used to exclude confounding non-cortical signals from the data (*hmrDeconvHRF_DriftSS*; trange -5.0 25.0, glmSolveMethod 1, idxBasis 2, paramsBasis 0.1 3.0 10.0 1.8 3.0 10.0, rhoSD_ssThresh 15.0, flagSSmethod 0, driftOrder 0, flagMotionCorrect 0) and block averages were created (*hmrBlockAvg*; trange -5.0 25.0).

The time courses of oxy-Hb and deoxy-Hb (hemodynamic response) from each participant were averaged for each task (fraction estimation and letter pairs condition, game-based and non-game-based task version, averaged across all item blocks each), respectively. Task-related concentration changes of oxy-Hb and deoxy-Hb were referred to a 5s baseline interval prior to item block onset (seconds -5 to 0). For statistical analyses, concentration changes of oxy-Hb and deoxy-Hb were averaged for the time period during which participants performed the respective item block (0–20 s after item block onset, this time span covered the average time needed by participants to complete the four items per block). In line with the NIRS study by Kober et al. [12], we analyzed the whole task period including number line estimation as well as feedback episodes.

### Pulse recordings and analysis

To measure participants’ heart rate, the portable 10-channel signal acquisition device NeXus-10 by MindMedia (Mind Media BV, Herten, The Netherlands) and a pulse sensor (PPG photoplethysmography), which was attached to the participant’s left index finger, were used. The NeXus-10 was connected to a computer via USB. All measurements were performed using the accompanying BioTrace+ software package (version V2012G1 Beta; Mind Media BV, Herten, The Netherlands).

All preprocessing steps were performed for both task versions separately (game-based, non-game-based) for each participant. The entire preprocessing procedure was run within Python 3.9. First, a high-pass filter at 0.5 Hz and a notch filter at 50 Hz were applied to the raw pulse data. Subsequently, peak detection was performed via the HeartPy library (version 1.2.7). By using a moving average and adaptive thresholds the HeartPy algorithm marks areas in the signal where all values surpass the respective threshold. Cardiac peaks are ultimately detected by determining the local maximum within a given area. For further information on the HeartPy library, please refer to [43]. Following that, instantaneous heart rate (IHR) was computed. In order to correct erroneous algorithmic actions, such as missing actual heart beats or incorrectly labelling noise spikes as cardiac peaks, outlier exclusion was performed by iterating over the IHR data twice and removing every IHR value five standard deviations above or below the respective mean [44]. Subsequently, mean task-related IHR was calculated. In addition, a task-related heart rate variability (HRV) measure was obtained by computing the standard deviation of all R-R intervals of heartbeats [45]. IHR and HRV values of each participant were averaged for each task (game-based and non-game-based task version of the fraction estimation condition, averaged across 12 item blocks each), respectively.

### Questionnaires

Affective states were assessed using the original German long version of the Multidimensional Mood State Questionnaire (original German title: “mehrdimensionaler Befindlichkeitsfragebogen”, MDBF) [46]. This self-report questionnaire has been constructed to evaluate the current mood state in various settings and includes 24 items. All items are singular adjectives that can be assigned to three bipolar subscales (eight items each): good-bad mood (“Gute-schlechte Stimmung”, α = .91 to .94, e.g., ‘content’), awake-tired (“Wachheit-Müdigkeit”, α = .92 to .94, e.g., ‘rested’), and calm-nervous (“Ruhe-Unruhe”, α = .86 to .91, e.g., ‘restless’). The items are answered using a 5-point Likert scale which describes the accuracy with which the item can be used to describe the current mood state. The answer scale ranges from ‘definitely not’ (“überhaupt nicht”) to ‘very much’ (“sehr”). For further data analysis, sums of the answers given in each subscale were calculated. These sums range from a minimum of 8 to a maximum of 40 possible points in each subscale.

To measure the participants’ flow experience, the Flow Short Scale (original German title: “Flow-Kurzskala”, FKS) was used [47]. The FKS has been constructed in accordance with Csikszentmihalyi’s flow theory [48] and is a self-report questionnaire including a total of 16 items. It can be used to measure the perceived fluency of a task (subscale “fluency”, 6 items, α = .92), immersion and absorption (subscale “absorption”, 4 items, α = .80), concern about the task (subscale “concern”, 3 items, α = .80 to .90) and the perceived fit of the task’s demands and the user’s skill (subscale “perceived fit of demands and skills”, 3 items). Items from the first three subscales are answered using a 7-point Likert scale ranging from 1 = strong disagreement (“trifft nicht zu”) to 7 = strong agreement with the statement (“trifft zu”). Example items for these scales are “*I don’t notice how much time is passing*” (item 3, subscale “absorption”) or “*I feel like I am in control of the task’s process*” (item 9, subscale “fluency”). The three items describing task demand, perceived skills, and the fit of these two measurements can be answered using 9-point Likert scales with answer options matching the specific item (e.g., the answer scale of item 15, “*I think my skills in this field are…*”, ranges from 1 = “low” to 9 = “high”). In addition to adding up the answers of the items per subscale, a general flow factor can also be obtained by summing up the responses to all items of the subscales “fluency” and “absorption”. These summed-up responses range from 6 to 42 for the subscale “fluency”, 4 to 28 for the subscale “absorption”, 10 to 70 for the general flow factor, and 9 to 21 for the subscales “concern” and “perceived fit of demands and skills”. Analyses of the participants’ flow experience in this study include the general flow factor and separate measures for each of the questionnaire subscales.

To evaluate participants’ perception of the game-based and non-game-based task, the German version of the User Experience Questionnaire (UEQ) was used [49]. The UEQ was developed specifically to assess users’ experience when interacting with different types of software, products, or digital interfaces. The UEQ is a self-report questionnaire consisting of 26 pairs of opposing adjectives (e.g., ‘unattractive’ and ‘attractive’). These items can be assigned to six subscales: Attractiveness (six items, α = .89, e.g., ‘annoying’ vs. ‘enjoyable’), Perspicuity (four items, α = .82, e.g., ‘not understandable’ vs. ‘understandable’), Dependability (four items, α = .65, e.g., ‘unpredictable’ vs. ‘predictable’), Efficiency (four items, α = .73, e.g., ‘fast’ vs. ‘slow’), Novelty (four items, α = .83, e.g., ‘creative’ vs. ‘dull’), and Stimulation (four items, α = .76, e.g., ‘boring’ vs. ‘exciting’). All UEQ items are answered on a 7-point Likert scale ranging from one adjective of the pair to the opposing one. For further analysis of the data, mean response scores were calculated for each of the six subscales using the UEQ data analysis tool [50]. These calculated means can reach a minimum of -3 and a maximum of 3 for each subscale.

### Procedure

At the beginning of the study, participants were asked to read and sign an informed consent form. Then, the experimenter explained the overall procedure of the study and instructed the participants on the goal, controls, and design of the game-based and non-game-based task version. Participants then completed a tutorial level of both task versions. The tutorial levels both consisted of one block of four fraction estimation items and one block of four letter pair items each followed by a 23 second break. Items used in the tutorial were not included in the subsequent tasks of the critical experiment. To prevent possible order effects, half of the participants completed the game-based condition first, while the other half started with the non-game-based condition. This task order counterbalancing was also balanced between the sexes of participants. Additionally, tutorials were presented in the same order as the conditions during the critical experiment. Following the tutorials, the NIRS cap was mounted on the participants’ head and the pulse sensor was affixed to the participant’s finger.

After completing the first task version, participants filled out the MDBF, FKS, and UEQ to assess their subjective experience during the previously completed task version. Then, the second task version started, which was again followed by the completion of the MDBF, FKS, and UEQ. The overall duration of the whole procedure was about 1.5 hours.

### Statistical analysis

Statistical analyses were performed in R 4.2.1 [51] and RStudio 2022.07.2 [52]. The R Code for all statistical analyses can be found in S1 and S2 Files. Tests for normal distribution can be found in S1 and S2 Files as well. Box plots were used to check for possible outliers (S1 File). Since excluding outliers (values larger or smaller 1.5 times the IQR) produced the same results as analyzing all data, we decided to analyze all data. For all statistical tests run, alpha level was set to *p* = 0.05.

To analyze the NIRS data, we performed mixed-effects models with the fixed effects *task-version* (game-based vs. non-game-based task version) and *hemisphere* (left vs. right), separately for oxy- and deoxy-Hb. Participants were included in the model as crossed random effects (see S1 File) [53]. For mixed-effects modeling, the R package *lme4* was used [54]. To display *F*-values (Type I Analysis of Variance with Satterthwaite’s method) the *lmertest* library was employed [55]. Additionally, paired *t*-tests comparing the game-based and the non-game-based task version per ROI were performed post hoc (see S1 File). These *t*-tests were performed in accordance with a previous NIRS study by Kober et al. [12] to achieve better comparability between studies.

Possible differences between the game- and the non-game-based task version in task performance (percentage of correct answers, accuracy), questionnaire data, and pulse recordings were analyzed using paired *t*-tests (see S2 File). Alpha level was adjusted using the procedure suggested by Holm [56] (S2 File).

## Results

### NIRS data

For oxy-Hb, the mixed-effects model revealed a significant main effect of *task-version* (*F*(1,120) = 5.1085, *p* = 0.0256, *ɳ*_*p*_^*2*^ = 2.36e-03). Oxy-Hb was higher in the game-based (*M* = 0.013 μM, *SD* = 0.06) than in the non-game-based task (*M* = -0.003 μM, *SD* = 0.064). The main effect of *hemisphere* (*F*(1,120) = 8.4188, *p* = 0.004419, *ɳ*_*p*_^*2*^ = 0.04) was significant, too, indicating a stronger oxy-Hb increase over the right (*M* = 0.015 μM, *SD* = 0.068) than over the left hemisphere (*M* = -0.005 μM, *SD* = 0.055). The interaction effect was not significant (*F*(1,120) = 1.7307, *p* = 0.190831).

For deoxy-Hb, the mixed-effect model revealed no significant results (main effect *task-version*: *F*(1,120) = 0.0801, *p* = 0.7777; main effect *hemisphere*: *F*(1,120) = 2.0116, *p* = 0.1587; interaction effect: *F*(1,120) = 0.0012, *p* = 0.9720).

**Fig 3:**
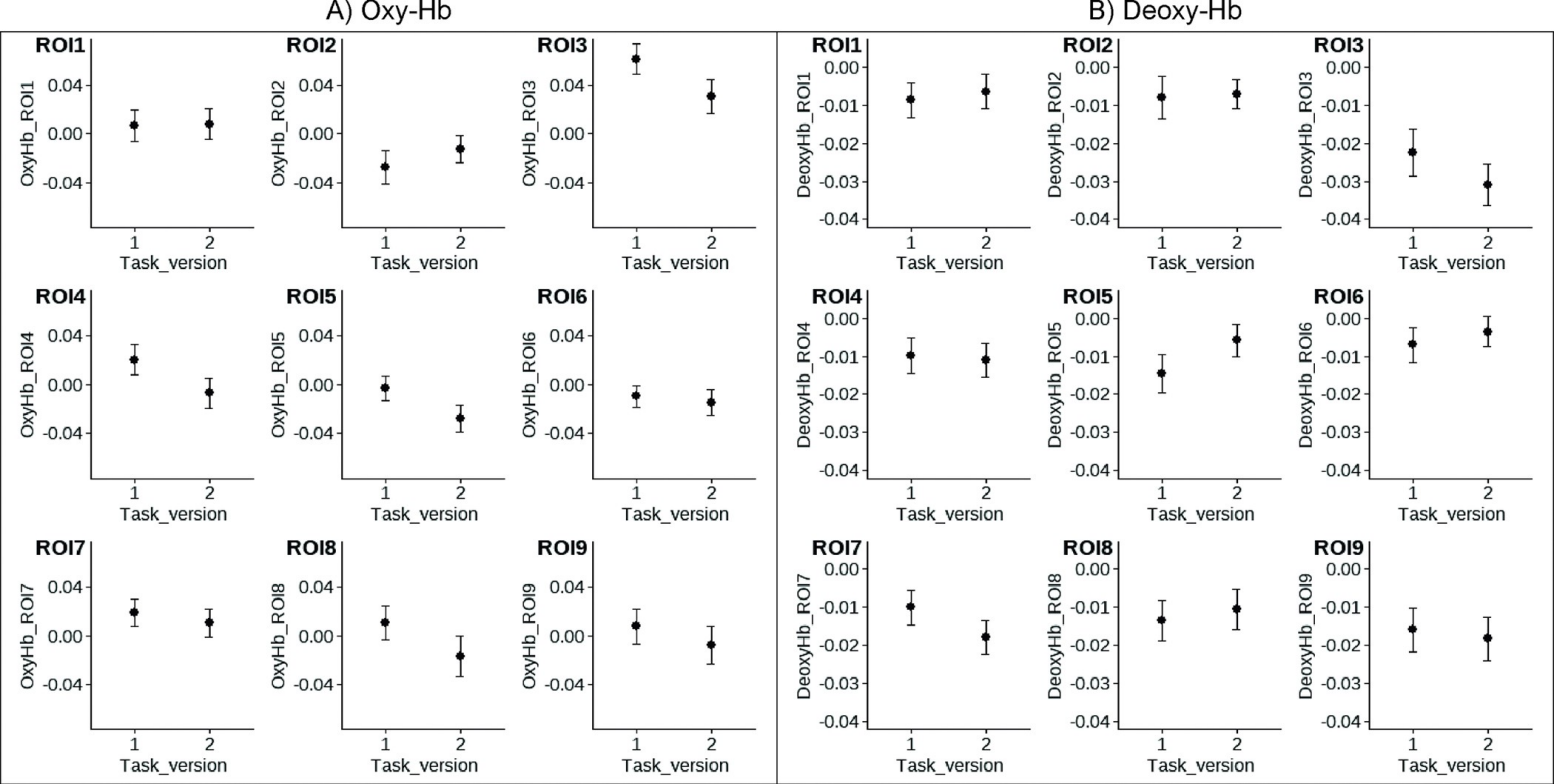
NIRS data per ROI. Means and *SE* of A) oxy- and B) deoxy-Hb [μM] for the game- (task version 1) and non-game-based task (task version 2) per region of interest (ROI). ROI 1: Superior frontal cortex right; ROI 2: Superior frontal cortex left; ROI 3: Middle frontal cortex right; ROI 4: Superior frontal cortex right; ROI5: Superior frontal cortex left and right; ROI 6: Superior frontal cortex left; ROI 7: Middle frontal cortex left; ROI 8: Orbitofrontal cortex right; ROI 9: Orbitofrontal cortex left.

**Fig 4:**
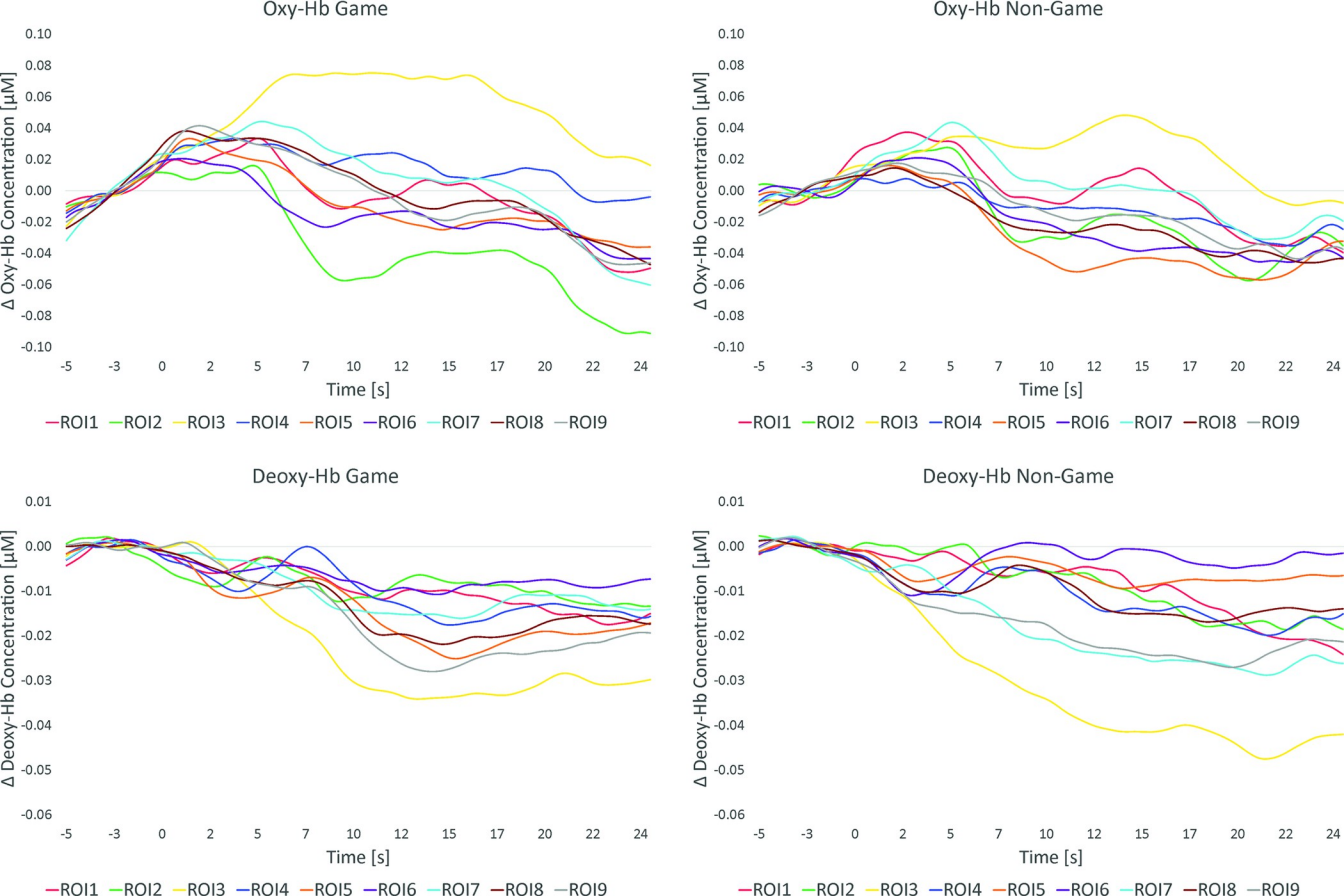
NIRS time courses. NIRS time course of oxy- (upper panel) and deoxy-Hb (lower panel) during the game-based (left panel) and non-game-based (right panel) task version, presented separately for each of the nine regions of interests (ROI).

**Fig 5:**
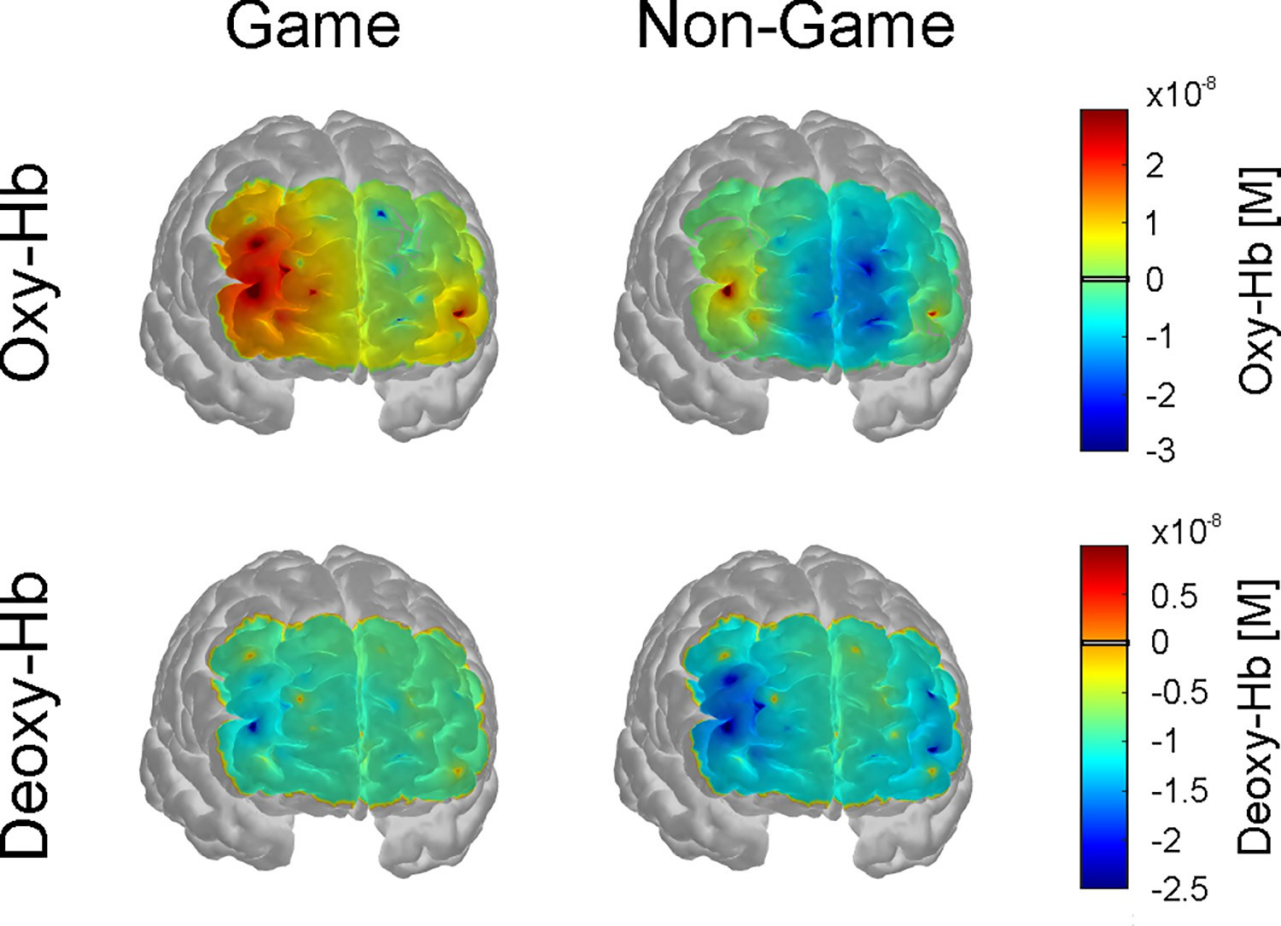
NIRS topography. Topographical distribution of mean oxy- and deoxy-Hb values (average of second 0–20 after task onset) during the game- and non-game-based task condition.

Table 1 summarizes the results of the pairwise comparisons of oxy- and deoxy-Hb between the game- and the non-game-based task condition per ROI. Fig 3 shows means and SE for oxy- and deoxy-Hb per ROI, separately for both task conditions. Fig 4 descriptively shows the NIRS time course during performing the game and the non-game-based task and Fig 5 shows the topographical distribution of the mean NIRS signal changes during task performance.

**Table 1:** Results of statistical analyses comparing oxy- and deoxy-Hb between the game- and the non-game-based task version separately per region of interest (ROI).

	Oxy-Hb	Deoxy-Hb
	Statistical result (effect size of significant results)	
ROI1 Superior frontal cortex right	*t*(40) = -0.10, *p* = 0.92	*t*(40) = -0.39, *p* = 0.70
ROI2 Superior frontal cortex left	*t*(40) = -0.85, *p* = 0.40	*t*(40) = -0.14, *p* = 0.89
ROI3 Middle frontal cortex right	*t*(40) = 2.51, *p* = 0.016* (*d* = 0.39)	*t*(40) = 1.40, *p* = 0.17
ROI4 Superior frontal cortex right	*t*(40) = 2.19, *p* = 0.034* (*d* = 0.34)	*t*(40) = 0.21, *p* = 0.83
ROI5 Superior frontal cortex left and right	*t*(40) = 2.52, *p* = 0.016* (*d* = 0.39)	*t*(40) = -1.77, *p* = 0.08
ROI6 Superior frontal cortex left	*t*(40) = 0.51, *p* = 0.61	*t*(40) = -0.71, *p* = 0.48
ROI7 Middle frontal cortex left	*t*(40) = 0.70, *p* = 0.49	*t*(40) = 1.59, *p* = 0.12
ROI8 Orbitofrontal cortex right	*t*(40) = 2.16, *p* = 0.037* (*d* = 0.34)	*t*(40) = -0.57, *p* = 0.57
ROI9 Orbitofrontal cortex left	*t*(40) = 1.35, *p* = 0.19	*t*(40) = 0.43, *p* = 0.67

Statistically significant *p*-values are marked with an asterisk. Alpha level was not adjusted for this explorative post-hoc analysis. *t*: statistical value for *t*-test; *p*: *p*-value; *d*: Cohen’s d.

### Task performance, user experience & physiological responses

Table 2 summarizes means and *SD*s of task performance and questionnaire data as well as the results of the statistical comparisons between the game-based and non-game-based task version.

**Table 2:** Task performance and user experience data (means and SD) and results of the statistical comparison between the game-based and non-game-based task version.

	Game		No-Game		
	*Mean*	*SD*	*Mean*	*SD*	*Statistical result (effect size for significant results)*
**Heart rate**					
IHR [bpm]	76.2	10.8	76.0	10.6	*t*(37) = 0.28, *p* = 0.78
HRV [ms]	71.9	21.1	75.4	24.7	*t*(37) = -0.95, *p* = 0.35
**Flow [raw values ranging from 10–70 (general factor), 6–42 (fluency), 4–28 (absorption), 9–21 (concern, perceived fit of demand and skills)]**					
General factor	46.5	8.18	44.0	7.72	*t*(40) = 1.73, *p* = 0.09
Fluency	31.9	5.76	30.6	6.10	*t*(40) = 1.26, *p* = 0.22
Absorption	14.7	4.37	13.4	3.42	*t*(40) = 2.00, *p* = 0.05
Concern	5.7	3.48	5.6	3.26	*t*(40) = -0.55, *p* = 0.59
Perceived fit of demands and skills	12.1	1.95	12.2	2.11	*t*(40) = -0.40, *p* = 0.69
**User Experience [raw values ranging from -3 to 3]**					
Attractiveness	1.23	0.88	0.08	1.09	*t*(40) = 5.53, *p* = 2.143e-06* (*d* = 0.86)
Perspicuity	2.20	0.97	2.24	0.95	*t*(40) = -0.23, *p* = 0.82
Efficiency	0.81	0.75	1.17	0.75	*t*(40) = -2.36, *p* = 0.02
Dependability	1.21	0.80	1.07	0.81	*t*(40) = 1.02, *p* = 0.31
Stimulation	0.23	1.01	-0.53	1.15	*t*(40) = 3.57, *p* = 0.0009* (*d* = 0.56)
Novelty	1.00	0.85	-0.84	1.12	*t*(40) = 7.77, *p* = 1.648e-09* (*d* = 1.21)
**Multidimensional Mood State Questionnaire [raw values ranging from 8 (bad mood, tired, nervous) to 40 (good mood, awake, calm)]**					
Good-bad mood	32.8	4.23	32.8	4.73	*t*(40) = 0.00, *p* = 1.00
Awake-tired	25.9	6.93	25.0	6.52	*t*(40) = 1.10, *p* = 0.28
Calm-nervous	32.2	5.33	32.7	4.46	*t*(40) = -0.80, *p* = 0.43
**Performance [%]**					
Accuracy	95.6	0.9	96.0	0.9	*t*(40) = -1.91, *p* = 0.06
Correct answers	93.3	4.47	94.0	4.32	*t*(40) = -0.76, *p* = 0.45

Statistically significant *p*-values are marked with an asterisk. For the analysis of the heart rate, only data of *N* = 38 participants were available. *t*: statistical value for *t*-test; *p*: *p*-value; *d*: Cohen’s d.

Task performance, mood, flow experience as well as heart rate did not differ between the task versions. According to the results of the UEQ, participants rated the game-based task version as significantly more attractive, stimulating, and novel.

## Discussion

In the present study, we aimed at expanding the findings of a previous fMRI study [13] about the neural correlates of game-based learning using NIRS, which is a more portable, flexible, and user-friendly neuroimaging technique compared to fMRI enabling the monitoring of brain activation patterns during gaming in a more ecologically valid and natural manner. Therefore, we compared the NIRS response over frontal brain areas between a game-based and a non-game-based version of a number line estimation task, the same task which was used by Greipl et al. [13]. While task performance, flow, and affective states were comparable between task conditions, the game-based task was rated as more attractive, stimulating, and novel compared to the non-game-based task and elicited a stronger frontal brain activation. In the following, we will first discuss results of brain activation patterns before we turn to results on performance, user experience, and physiological responses.

### Brain activation

Overall, the game-based task version led to a stronger increase in oxy-Hb than the non-game-based task version over the frontal areas assessed by the current NIRS setup. This is in line with previous evidence indicating that estimating numbers or fractions on a number line seems to generally activate a fronto-parietal network [57]. In this context, it is assumed that activation in frontal areas not only reflects number processing but also non-numerical processes such as attention [for a meta-analysis see 58]. Hence, the stronger frontal activation while performing the game-based task might be related to a stronger attentional engagement compared to the non-game-based task [12].

Additionally, activation in the right frontal cortex was stronger during task completion independent of the task version. This is in line with prior fMRI and NIRS studies showing that number line estimation leads to stronger activation of the right frontal cortex including the superior and middle frontal gyrus compared to the left frontal cortex [12, 57].

On an exploratory level, when having a closer look at the specific regions of interest (ROI), the right middle frontal cortex (ROI3), the right superior frontal cortex (ROI 4), and the right orbitofrontal cortex (ROI 8) were activated more strongly during the game-based compared to the non-game-based task.

More pronounced activation in the orbitofrontal cortex might reflect increased processing of reward and emotional aspects of the game-based task version [15, 59–61]. This is in line with previous evidence indicating that the inclusion of game elements in cognitive tasks as well as learning tasks can modulate emotional states [31, 62–66]. In the present study, feedback and rewards for correct and incorrect number line estimations were presented in a more contextualized and emotion-inducing way in the game-based task version. In particular, while in the non-game-based task feedback was only presented in the form of a green tick or a red X (Fig 1), in the game this feedback was extended with emotional reactions of the game avatar, i.e., avatar was smiling or crying (Fig 1). Greipl et al. [13], who used the same task versions in an fMRI study, also found significantly stronger activation of the orbitofrontal cortex during the game- compared to the non-game-based task and linked this result to differences in emotional and reward processing.

Stronger activation in the right superior frontal cortex might be caused by the richer sensory stimulation in the game-based task version compared to the non-game-based version. Fuster et al. [67] argues that the superior frontal cortex is, among other things, activated by sensory stimuli like visual information. The game-based task features more and also more diverse visual information than the non-game-based task (Fig 1), which might thus have increased activation in the right superior frontal cortex.

Higher activation of the middle frontal cortex was previously linked to increased working memory load and different attentional processes during the game-based task compared to simpler non-game-based tasks containing less complex visual design [12]. This might suggest that the game elements increased unnecessary, extraneous cognitive processing as suggested by cognitive theories on multimedia learning [68]. According to these theories, irrelevant game elements can distract learners from the essential learning material. However, we did not find any difference in performance between the game and non-game-based task (see below). The performance of the participants was very high, which may indicate that the possible extraneous processing did not overload participants’ cognitive capacity, and thus the risk of disturbing performance was low. On the other hand, it is questionable whether the game elements can be considered as irrelevant as the included elements were intrinsically integrated with the core learning mechanic.

No significant changes in deoxy-Hb concentration were found. Generally, oxy-Hb showed more pronounced concentration changes than deoxy-Hb [38]. Additionally, oxy-Hb is a more sensitive indicator for changes in cerebral blood flow than deoxy-Hb [69]. Therefore, it is relatively common in NIRS studies to observe significant changes in oxy- but not in deoxy-Hb.

### Task performance

We observed no differences in task performance between both task versions. Previous studies investigating neural correlates of game- vs. non-game-based versions of a number line estimation task also reported no significant differences between task version in the number of correct answers [12, 13]. However, Greipl et al. [13] observed significantly higher accuracy of fraction estimation in the non-game-based task compared to the game-based task. Generally, task performance of the present sample of healthy adults was quite high even though we adapted the used items by using more difficult fractions (i.e., fractions with numerators and denominators ranging from 2 to 29). Nevertheless, in the present study, participants administered only one playing session and thus improvements were not expected. Previous studies reporting on performance improvements when playing game-based rational number training performed multiple training sessions [11].

In some publications, concerns were raised about the potential detrimental effects of the use of game elements in learning tasks. For instance, it was found that information that captures attention but is irrelevant to the task at hand can interfere with learning [seductive detail effect; e.g., 70, 71] by increasing extraneous processing demands [68]. Accordingly, immersive game-based environments, which are rich in details, can lead to poorer learning outcomes. This relationship might be caused by increased cognitive/mental load in the more complex game-based environment [72]. In this context, the current result that task performance did not differ between a more detailed and visually rich game-based task and a very simplistic, non-game-based one also indicates that differences in frontal brain activation between task versions might not be related to differences in task performance, mental load, or task difficulty [12, 32, 73]. Accordingly, one might also speculate that game elements induce different attentional processes or strategies, rather than only influencing extraneous processing demands–at least when incorporating the game elements in an intrinsically integrated way [6, 28, 74]. We cannot exclude that game elements increased extraneous processing in the game-based task version, however, the results of the task performance indicate that such a possible extraneous processing did not lead to detrimental effects on performance. Furthermore, the high task performance of the present sample may mask more pronounced effects of interference of game elements on task performance. This is to be investigated in follow-up studies.

### User experience

In line with previous studies, participants rated the game-based task version as more attractive, stimulating and novel [12, 13, 32]. Perceived aesthetics of a game-based learning task can affect emotional states and consequently intrinsic motivation of learners [31, 62, 75]. This in turn is in line with our findings of more pronounced activation of the frontal cortex involved in emotional processing and attention. Future studies might also investigate how user experience is affected by gender and personality in game-based learning [76].

Flow experience was comparable in both task versions. Prior studies reported heterogeneous results concerning the effects of game elements on flow experience [e.g., 12, 31, 77]. Generally, it is assumed that a state of flow can only be reached when engaging with a task that is somewhat challenging [48]. In this study, participants’ performance was rather high in both conditions. Thus, one may argue that both task versions might not have been challenging enough for participants to actually reach a flow state. Furthermore, the simple design of the game, lack of a clear overarching goal and possibility to fail in the game (e.g., lack of virtual energy metrics) may have lowered the possibility to reach a flow state. The timing of the task (short item blocks followed by breaks) may also have hindered the emergence of a strong flow experience. Participants were frequently interrupted in their workflow and left idle for 23 seconds during the breaks before they could return to their tasks of fraction estimation. It is questionable whether such a structure is beneficial for the development of flow since the flow experience is dependent on a person’s possibility to engage with a task [48].

Participants also reported comparable mood levels in both task conditions. Prior studies found a higher level of joy reported by participants during a game-based compared to a non-game-based task version [12, 78]. Yet, in the studies by Kober et al. [12] and Brom et al. [78], a different questionnaire was used to assess affective states during task performance, namely the Positive and Negative Affect Schedule (PANAS). Hence, results seem not directly comparable. Furthermore, the game and non-game-based version in the study by Brom et al. [78] differed on more levels than on visual appearance and emotionally rich feedback (e.g. presence or absence of competition). Taken together, our results suggest that using only game elements that are integrated to the core learning mechanic does not create such a gameful experience that facilitates enjoyment.

### Physiological responses

We also assessed physiological responses (e.g., heart rate) while participants performed the task as differences in emotional engagement between task versions might also affect them [33, 34]. However, no differences in heart rate between task versions were observed. Increases in heart rate during gaming are mostly dependent on the choice of game and the game setting [33]. Studies reporting increases in heart rate typically investigated computer games of genres such as survival horror, adventure, sports, or fighting. The game-based task of the present study included relatively simple control patterns, slow pacing, relatively simple goals, and weak negative consequences of failures, possibly leading to lower arousal than found in the studies reviewed by [33]. Furthermore, there is evidence that, in particular, tasks requiring high cognitive effort lead to emotional arousal followed by an increase in heart rate [79]. Yet, as the current task of fraction estimation, task controls, and the goals of the present game- and non-game-based task version as well as task performance were (observed to be) comparable, cognitive demands to perform both tasks might be too similar to elicit significant differences in physiological responses. Additionally, no significant differences in the participants’ mood or their concern regarding the task at hand (flow subfactor “Concern” of the FKS) were found between the two task conditions. In turn, this substantiates our interpretation that emotional arousal elicited by the game- and the non-game-based task may be seen as comparable and may thus also lead to similar physiological responses.

## Conclusions

In the current study, we replicated previous findings of increased frontal brain activation observed for a game-based version of a fraction learning task compared to a non-game-based control task using NIRS. It is important to note that we used a simple game-based learning task in which the core learning mechanic (number line estimation) was intrinsically integrated with only a limited number of game elements. We did not include other common game elements that are used to create tension and a more immersive playing experience to be able to investigate the effects of the core gameplay in more detail. While adding game elements to the learning task had no effects on task performance within the session, game elements seemed to activate brain areas associated with reward processing, emotional engagement and attention. Furthermore, the game-based task version led to higher ratings of attractiveness, stimulation, and novelty compared to a non-game-based task version. These factors might have a positive effect on learning in case such a game-based task is used over a longer period of time. This needs to be further investigated in future training studies.

## Supporting information

S1 File NIRS analysis code. RMarkdown script for the analysis of the NIRS data.
(HTML)

S2 File Behavioral analysis code. RMarkdown script for the analysis of the behavioral data, i.e., task performance, user experience, etc.
(HTML)

S3 File NIRS data. Data for the NIRS analysis.
(TXT)

S4 File Behavioral data. Data for the behavioral analysis.
(TXT)
