# Supplementary material for: Engaging learners with games–Insights from functional near-infrared spectroscopy
Source: PLoS One. 2023 Jun 6;18(6):e0286450. doi: 10.1371/journal.pone.0286450 (PMC10243642; doi:10.1371/journal.pone.0286450)
Supplement: S1 File — RMarkdown script for the analysis of the NIRS data. (HTML) [file pone.0286450.s001.html]

Supplementary Material S1: R Code for statistical analysis of NIRS data


# Supplementary Material S1: R Code for statistical analysis of NIRS data

#### 2022-12-12

This RMarkdown shows the analysis of the NIRS data (oxy- and
deoxy-Hb) using linear mixed‐effects models and t-tests using R (version
4.2.1).

## Loading R packages and importing dataset

```
library(lme4) # for calculating linear mixed-effects models
library(emmeans) # for performing post-tests for sign. interactions
library(lmerTest) # Tests in linear mixed-effects models
library(dlookr) # for checking distribution of the dataset
library(sjstats) # for anova + partial eta squared
library(tidyverse) # for cleaning up data / plotting
library(dplyr) # to manipulate data set
library(ggpubr) # to plot data
library(PairedData) # for paired data analysis
library(coin) # for calculating effect sizes Wilcoxon tests
library(rstatix) # for calculating effect sizes t-tests
library(moments) #  skewness and kurtosis, testing whether skewness and kurtosis match a normal distribution
```

```
# set working directory, where the S3_File.txt file is stored
setwd("E:/")
myData <- read.table("S3_File.txt", header = T, dec = ".")
head(myData)
```

```
##   Code Task_version   OxyHb_ROI1  OxyHb_ROI2   OxyHb_ROI3   OxyHb_ROI4
## 1    1            1 -0.052760123 -0.02631760 -0.058797389 -0.005772604
## 2    3            1 -0.109723430 -0.20163303  0.201724797 -0.024277747
## 3    4            1  0.007015618 -0.08572154  0.067636099 -0.035539106
## 4    5            1  0.021611627 -0.16155239  0.155866575 -0.072615513
## 5    6            1 -0.046371509  0.03906104  0.004535641 -0.031841090
## 6    7            1 -0.030204822 -0.04897816  0.020741647 -0.146153381
##     OxyHb_ROI5  OxyHb_ROI6   OxyHb_ROI7   OxyHb_ROI8  OxyHb_ROI9    OxyHb_LH
## 1 -0.072335144 -0.01362036 -0.000619722 -0.005033755 -0.07850978 -0.02848677
## 2 -0.143592643 -0.06297816  0.030010772 -0.046631735 -0.07773282 -0.07965303
## 3  0.002606494  0.02202704 -0.040385500 -0.081620458 -0.02907955 -0.02369532
## 4 -0.164209544  0.00182437  0.091568607 -0.070322056 -0.10468514 -0.05278227
## 5  0.005263492  0.02994313 -0.012067812 -0.042369575 -0.01533492  0.01042558
## 6 -0.114691793 -0.10413121 -0.003291699 -0.063399329 -0.02726324 -0.05265686
##       OxyHb_RH  DeoxyHb_ROI1 DeoxyHb_ROI2 DeoxyHb_ROI3 DeoxyHb_ROI4
## 1 -0.033468433  0.0041953020 -0.001229109 -0.019313769 -0.023992184
## 2  0.008737147 -0.0149760600 -0.037983924 -0.095926323  0.002533169
## 3 -0.002950483 -0.0240799160 -0.006393497 -0.029245478 -0.022004181
## 4  0.013701843  0.0123929630  0.050823451 -0.105055579  0.015488610
## 5 -0.025815097 -0.0000554138 -0.003089568  0.004837476 -0.007770028
## 6 -0.064119281  0.0056343990 -0.004551850 -0.026387666  0.030159844
##   DeoxyHb_ROI5 DeoxyHb_ROI6 DeoxyHb_ROI7 DeoxyHb_ROI8 DeoxyHb_ROI9   DeoxyHb_LH
## 1 -0.009543281 -0.005473643 -0.017657554 -0.016500885 -0.007104222 -0.008670535
## 2 -0.011292661 -0.030472446 -0.061492161  0.011261100 -0.060678149 -0.044785009
## 3 -0.038244388 -0.021091383 -0.023159517  0.003606280 -0.008103639 -0.018917523
## 4 -0.044049657 -0.019072980 -0.055882642  0.037272897  0.009802722 -0.021105805
## 5 -0.016503848 -0.021839898  0.001755461 -0.007917158 -0.007370578 -0.008031040
## 6  0.064379103  0.042052157 -0.004663734  0.046376092  0.063948310  0.024014855
##     DeoxyHb_RH
## 1 -0.013751361
## 2 -0.030857683
## 3 -0.020140796
## 4 -0.020644943
## 5 -0.003739608
## 6  0.010719410
```

```
# Code: Participant code
# Task_version: 1 = Game-based task version, 2 = Non-game-based task version
# OxyHb_ROI1: mean oxygenated hemoglobin (aveage of second 0-20 afer task onset) during task processing for NIRS channels forming ROI1
# ....
# OxyHb_ROI9: mean oxygenated hemoglobin (aveage of second 0-20 afer task onset) during task processing for NIRS channels forming ROI9
# OxyHb_LH: mean oxygenated hemoglobin (aveage of second 0-20 afer task onset) during task processing for NIRS channels over the left hemisphere
# OxyHb_RH: mean oxygenated hemoglobin (aveage of second 0-20 afer task onset) during task processing for NIRS channels over the right hemisphere
# DeoxyHb_ROI1: mean deoxygenated hemoglobin (aveage of second 0-20 afer task onset) during task processing for NIRS channels forming ROI1
# ...
# DeoxyHb_ROI9: mean deoxygenated hemoglobin (aveage of second 0-20 afer task onset) during task processing for NIRS channels forming ROI9
# DeoxyHb_LH: mean deoxygenated hemoglobin (aveage of second 0-20 afer task onset) during task processing for NIRS channels over the left hemisphere
# DeoxyHb_RH: mean deoxygenated hemoglobin (aveage of second 0-20 afer task onset) during task processing for NIRS channels over the right hemisphere
```

## Prepare dataset for analyzing oxy-Hb

```
myLongFormat <- myData[, c(1, 2, 12 )]

for(i in 1:1){
i <- i + 12
colnames(myData)[c(1, 2, i)] <- colnames(myLongFormat)
myLongFormat <- rbind(myLongFormat, myData[, c(1, 2, i)], make.row.names = F)
}

head(myLongFormat)
```

```
##   Code Task_version    OxyHb_LH
## 1    1            1 -0.02848677
## 2    3            1 -0.07965303
## 3    4            1 -0.02369532
## 4    5            1 -0.05278227
## 5    6            1  0.01042558
## 6    7            1 -0.05265686
```

```
hemisphere <- as.numeric(gl(2, dim(myData)[1]))
myLongFormat <- cbind(myLongFormat, as.numeric(substr(myLongFormat[, 1], 1, 2)), hemisphere)
colnames(myLongFormat)[c(4)] <- "pb"
colnames(myLongFormat)[c(3)] <- "oxy_Hb"

head(myLongFormat) # hemisphere: 1 = left, 2 = right
```

```
##   Code Task_version      oxy_Hb pb hemisphere
## 1    1            1 -0.02848677  1          1
## 2    3            1 -0.07965303  3          1
## 3    4            1 -0.02369532  4          1
## 4    5            1 -0.05278227  5          1
## 5    6            1  0.01042558  6          1
## 6    7            1 -0.05265686  7          1
```

## Linear mixed‐effects‐models (LMM) for oxy-Hb

Now we calculate the linear mixed‐effects model for oxy-Hb

```
m1  <- lmer(oxy_Hb ~ Task_version*hemisphere +(1|pb), myLongFormat, na.action = na.omit )

anova(m1, type = "I", ddf = "Satterthwaite")
```

```
## Type I Analysis of Variance Table with Satterthwaite's method
##                            Sum Sq   Mean Sq NumDF DenDF F value   Pr(>F)   
## Task_version            0.0102412 0.0102412     1   120  5.1085 0.025612 * 
## hemisphere              0.0168774 0.0168774     1   120  8.4188 0.004419 **
## Task_version:hemisphere 0.0034696 0.0034696     1   120  1.7307 0.190831   
## ---
## Signif. codes:  0 '***' 0.001 '**' 0.01 '*' 0.05 '.' 0.1 ' ' 1
```

Descriptive statistics: Means and SD of data

```
myLongFormat %>%
  group_by(Task_version, hemisphere) %>%
  get_summary_stats(oxy_Hb, type = "mean_sd")
```

```
## # A tibble: 4 × 6
##   Task_version hemisphere variable     n   mean    sd
##          <int>      <dbl> <chr>    <dbl>  <dbl> <dbl>
## 1            1          1 oxy_Hb      41 -0.002 0.053
## 2            1          2 oxy_Hb      41  0.028 0.063
## 3            2          1 oxy_Hb      41 -0.009 0.057
## 4            2          2 oxy_Hb      41  0.003 0.071
```

```
myLongFormat %>%
  group_by(Task_version) %>%
  get_summary_stats(oxy_Hb, type = "mean_sd")
```

```
## # A tibble: 2 × 5
##   Task_version variable     n   mean    sd
##          <int> <chr>    <dbl>  <dbl> <dbl>
## 1            1 oxy_Hb      82  0.013 0.06 
## 2            2 oxy_Hb      82 -0.003 0.064
```

```
myLongFormat %>%
  group_by(hemisphere) %>%
  get_summary_stats(oxy_Hb, type = "mean_sd")
```

```
## # A tibble: 2 × 5
##   hemisphere variable     n   mean    sd
##        <dbl> <chr>    <dbl>  <dbl> <dbl>
## 1          1 oxy_Hb      82 -0.005 0.055
## 2          2 oxy_Hb      82  0.015 0.068
```

Checking assumptions

```
residuals_m1 <- resid(m1)
fitted_m1 <- fitted(m1)

plot(residuals_m1[1:41] ~ fitted_m1[1:41], main = "Residuals - Game, left hemisphere")
```

```
qqnorm(residuals_m1[1:41], main = "QQ-Plot - Game, left hemisphere")
qqline(residuals_m1[1:41])
```

```
jarque.test(residuals_m1[1:41])
```

```
## 
##  Jarque-Bera Normality Test
## 
## data:  residuals_m1[1:41]
## JB = 0.45186, p-value = 0.7978
## alternative hypothesis: greater
```

```
plot(residuals_m1[42:82] ~ fitted_m1[42:82], main = "Residuals - Non-Game, left hemisphere")
```

```
qqnorm(residuals_m1[42:82], main = "QQ-Plot - Non-Game, left hemisphere")
qqline(residuals_m1[42:82])
```

```
jarque.test(residuals_m1[42:82])
```

```
## 
##  Jarque-Bera Normality Test
## 
## data:  residuals_m1[42:82]
## JB = 3.6413, p-value = 0.1619
## alternative hypothesis: greater
```

```
plot(residuals_m1[83:123] ~ fitted_m1[83:123], main = "Residuals - Game, right hemisphere")
```

```
qqnorm(residuals_m1[83:123], main = "QQ-Plot - Game, right hemisphere")
qqline(residuals_m1[83:123])
```

```
jarque.test(residuals_m1[83:123])
```

```
## 
##  Jarque-Bera Normality Test
## 
## data:  residuals_m1[83:123]
## JB = 0.85724, p-value = 0.6514
## alternative hypothesis: greater
```

```
plot(residuals_m1[124:164] ~ fitted_m1[124:164], main = "Residuals - Non-Game, right hemisphere")
```

```
qqnorm(residuals_m1[124:164], main = "QQ-Plot - Non-Game, right hemisphere")
qqline(residuals_m1[124:164])
```

```
jarque.test(residuals_m1[124:164])
```

```
## 
##  Jarque-Bera Normality Test
## 
## data:  residuals_m1[124:164]
## JB = 14.409, p-value = 0.0007431
## alternative hypothesis: greater
```

According to the Jarque-Bera Normality Test, skewness and kurtosis of
the data match a normal distribution except for the non-game condition -
right hemisphere. Therefore, in the next step we check the data for
possible outliers.

Checking for outliers using Box-Plots

```
ggplot(myLongFormat, aes(factor(Task_version), oxy_Hb, fill = factor(hemisphere))) + geom_boxplot() +
    scale_fill_brewer(palette = "Dark2",
    name = "Hemisphere",
    labels =    c("left", "right")) +
    theme_classic() +
    scale_x_discrete(name = "Game condition",
    labels = c("Game", "Non-Game")) +
    scale_y_continuous(name = "Oxy Hb [micromol]")
```

We see that there are potential outliers (values larger or smaller
1.5 times the IQR) in the non-game condition. Let’s exclude these
outlier values and recalculate the mixed-effects model to see if results
would change.

```
Game_LH <- subset(myLongFormat[myLongFormat$Task_version == 1 & myLongFormat$hemisphere == 1, ], )

# create a vector of outliers for the numeric factor
outliers_Game_LH <- boxplot(Game_LH$oxy_Hb, plot = FALSE)$out

# Replace the values with NA
Game_LH[Game_LH$oxy_Hb %in% outliers_Game_LH, "oxy_Hb"] = NA

Game_RH <- subset(myLongFormat[myLongFormat$Task_version == 1 & myLongFormat$hemisphere == 2, ], )

# create a vector of outliers for the numeric factor
outliers_Game_RH <- boxplot(Game_RH$oxy_Hb, plot = FALSE)$out

# Replace the values with NA
Game_RH[Game_RH$oxy_Hb %in% outliers_Game_RH, "oxy_Hb"] = NA

NoGame_LH <- subset(myLongFormat[myLongFormat$Task_version == 2 & myLongFormat$hemisphere == 1, ], )

# create a vector of outliers for the numeric factor
outliers_NoGame_LH <- boxplot(NoGame_LH$oxy_Hb, plot = FALSE)$out

# Replace the values with NA
NoGame_LH[NoGame_LH$oxy_Hb %in% outliers_NoGame_LH, "oxy_Hb"] = NA

NoGame_RH <- subset(myLongFormat[myLongFormat$Task_version == 2 & myLongFormat$hemisphere == 2, ], )

# create a vector of outliers for the numeric factor
outliers_NoGame_RH <- boxplot(NoGame_RH$oxy_Hb, plot = FALSE)$out

# Replace the values with NA
NoGame_RH[NoGame_RH$oxy_Hb %in% outliers_NoGame_RH, "oxy_Hb"] = NA

myLongFormat_noOutliers <- rbind(Game_LH, Game_RH, NoGame_LH, NoGame_RH)

m1_noOutliers  <- lmer(oxy_Hb ~ Task_version*hemisphere +(1|pb), myLongFormat_noOutliers, na.action = na.omit )
anova(m1_noOutliers, type = "I", ddf = "Satterthwaite")
```

```
## Type I Analysis of Variance Table with Satterthwaite's method
##                            Sum Sq   Mean Sq NumDF  DenDF F value    Pr(>F)    
## Task_version            0.0114452 0.0114452     1 113.76  7.6717 0.0065526 ** 
## hemisphere              0.0178179 0.0178179     1 113.01 11.9433 0.0007735 ***
## Task_version:hemisphere 0.0027906 0.0027906     1 113.07  1.8706 0.1741228    
## ---
## Signif. codes:  0 '***' 0.001 '**' 0.01 '*' 0.05 '.' 0.1 ' ' 1
```

Results are the same with and without exclusion of outliers.
Therefore, we decided to report on the analysis of the whole data set in
the manuscript.

## T-Tests per ROI for oxy-Hb

```
setwd("E:/")

myData2 <- read.table("S3_File.txt", header = T, dec = ".")
head(myData2)
```

```
##   Code Task_version   OxyHb_ROI1  OxyHb_ROI2   OxyHb_ROI3   OxyHb_ROI4
## 1    1            1 -0.052760123 -0.02631760 -0.058797389 -0.005772604
## 2    3            1 -0.109723430 -0.20163303  0.201724797 -0.024277747
## 3    4            1  0.007015618 -0.08572154  0.067636099 -0.035539106
## 4    5            1  0.021611627 -0.16155239  0.155866575 -0.072615513
## 5    6            1 -0.046371509  0.03906104  0.004535641 -0.031841090
## 6    7            1 -0.030204822 -0.04897816  0.020741647 -0.146153381
##     OxyHb_ROI5  OxyHb_ROI6   OxyHb_ROI7   OxyHb_ROI8  OxyHb_ROI9    OxyHb_LH
## 1 -0.072335144 -0.01362036 -0.000619722 -0.005033755 -0.07850978 -0.02848677
## 2 -0.143592643 -0.06297816  0.030010772 -0.046631735 -0.07773282 -0.07965303
## 3  0.002606494  0.02202704 -0.040385500 -0.081620458 -0.02907955 -0.02369532
## 4 -0.164209544  0.00182437  0.091568607 -0.070322056 -0.10468514 -0.05278227
## 5  0.005263492  0.02994313 -0.012067812 -0.042369575 -0.01533492  0.01042558
## 6 -0.114691793 -0.10413121 -0.003291699 -0.063399329 -0.02726324 -0.05265686
##       OxyHb_RH  DeoxyHb_ROI1 DeoxyHb_ROI2 DeoxyHb_ROI3 DeoxyHb_ROI4
## 1 -0.033468433  0.0041953020 -0.001229109 -0.019313769 -0.023992184
## 2  0.008737147 -0.0149760600 -0.037983924 -0.095926323  0.002533169
## 3 -0.002950483 -0.0240799160 -0.006393497 -0.029245478 -0.022004181
## 4  0.013701843  0.0123929630  0.050823451 -0.105055579  0.015488610
## 5 -0.025815097 -0.0000554138 -0.003089568  0.004837476 -0.007770028
## 6 -0.064119281  0.0056343990 -0.004551850 -0.026387666  0.030159844
##   DeoxyHb_ROI5 DeoxyHb_ROI6 DeoxyHb_ROI7 DeoxyHb_ROI8 DeoxyHb_ROI9   DeoxyHb_LH
## 1 -0.009543281 -0.005473643 -0.017657554 -0.016500885 -0.007104222 -0.008670535
## 2 -0.011292661 -0.030472446 -0.061492161  0.011261100 -0.060678149 -0.044785009
## 3 -0.038244388 -0.021091383 -0.023159517  0.003606280 -0.008103639 -0.018917523
## 4 -0.044049657 -0.019072980 -0.055882642  0.037272897  0.009802722 -0.021105805
## 5 -0.016503848 -0.021839898  0.001755461 -0.007917158 -0.007370578 -0.008031040
## 6  0.064379103  0.042052157 -0.004663734  0.046376092  0.063948310  0.024014855
##     DeoxyHb_RH
## 1 -0.013751361
## 2 -0.030857683
## 3 -0.020140796
## 4 -0.020644943
## 5 -0.003739608
## 6  0.010719410
```

Plot mean and SE per ROI and task condition.

```
ROI1_mean <- ggerrorplot(myData2, x = "Task_version", y = "OxyHb_ROI1", 
            desc_stat = "mean_se",
            ylim = c(-0.07, 0.07),
            error.plot = "errorbar",            # Change error plot type
            add = "mean"                        # Add mean points
            )

ROI2_mean <- ggerrorplot(myData2, x = "Task_version", y = "OxyHb_ROI2", 
            desc_stat = "mean_se",
            error.plot = "errorbar",            # Change error plot type
            ylim = c(-0.07, 0.07),
                add = "mean"                        # Add mean points
            )

ROI3_mean <- ggerrorplot(myData2, x = "Task_version", y = "OxyHb_ROI3", 
            desc_stat = "mean_se",
            error.plot = "errorbar",            # Change error plot type
            ylim = c(-0.07, 0.07),
                add = "mean"                        # Add mean points
            )

ROI4_mean <- ggerrorplot(myData2, x = "Task_version", y = "OxyHb_ROI4", 
            desc_stat = "mean_se",
            error.plot = "errorbar",            # Change error plot type
            ylim = c(-0.07, 0.07),
                add = "mean"                        # Add mean points
            )


ROI5_mean <- ggerrorplot(myData2, x = "Task_version", y = "OxyHb_ROI5", 
            desc_stat = "mean_se",
            error.plot = "errorbar",            # Change error plot type
            ylim = c(-0.07, 0.07),
                add = "mean"                        # Add mean points
            )

ROI6_mean <- ggerrorplot(myData2, x = "Task_version", y = "OxyHb_ROI6", 
            desc_stat = "mean_se",
            error.plot = "errorbar",            # Change error plot type
            ylim = c(-0.07, 0.07),
                add = "mean"                        # Add mean points
            )

ROI7_mean <- ggerrorplot(myData2, x = "Task_version", y = "OxyHb_ROI7", 
            desc_stat = "mean_se",
            error.plot = "errorbar",            # Change error plot type
            ylim = c(-0.07, 0.07),
                add = "mean"                        # Add mean points
            )

ROI8_mean <- ggerrorplot(myData2, x = "Task_version", y = "OxyHb_ROI8", 
            desc_stat = "mean_se",
            error.plot = "errorbar",            # Change error plot type
            ylim = c(-0.07, 0.07),
                add = "mean"                        # Add mean points
            )

ROI9_mean <- ggerrorplot(myData2, x = "Task_version", y = "OxyHb_ROI9", 
            desc_stat = "mean_se",
            error.plot = "errorbar",            # Change error plot type
            ylim = c(-0.07, 0.07),
                add = "mean"                        # Add mean points
            )

ggarrange(ROI1_mean, ROI2_mean, ROI3_mean, ROI4_mean, ROI5_mean, ROI6_mean,
        ROI7_mean, ROI8_mean, ROI9_mean,
         labels = c("ROI1", "ROI2", "ROI3", "ROI4", "ROI5", "ROI6", "ROI7", "ROI8", "ROI9"),
           ncol = 3, nrow = 3)
```

Check normal distribution.

```
# compute the difference
d1 <- with(myData2, OxyHb_ROI1[Task_version == "1"] - OxyHb_ROI1[Task_version == "2"])
# Shapiro-Wilk normality test for the differences
shapiro.test(d1)
```

```
## 
##  Shapiro-Wilk normality test
## 
## data:  d1
## W = 0.96911, p-value = 0.3224
```

```
d2 <- with(myData2, OxyHb_ROI2[Task_version == "1"] - OxyHb_ROI2[Task_version == "2"])
shapiro.test(d2)
```

```
## 
##  Shapiro-Wilk normality test
## 
## data:  d2
## W = 0.97579, p-value = 0.521
```

```
d3 <- with(myData2, OxyHb_ROI3[Task_version == "1"] - OxyHb_ROI3[Task_version == "2"])
shapiro.test(d3)
```

```
## 
##  Shapiro-Wilk normality test
## 
## data:  d3
## W = 0.96762, p-value = 0.2878
```

```
d4 <- with(myData2, OxyHb_ROI4[Task_version == "1"] - OxyHb_ROI4[Task_version == "2"])
shapiro.test(d4)
```

```
## 
##  Shapiro-Wilk normality test
## 
## data:  d4
## W = 0.90871, p-value = 0.003027
```

```
d5 <- with(myData2, OxyHb_ROI5[Task_version == "1"] - OxyHb_ROI5[Task_version == "2"])
shapiro.test(d5)
```

```
## 
##  Shapiro-Wilk normality test
## 
## data:  d5
## W = 0.94787, p-value = 0.05895
```

```
d6 <- with(myData2, OxyHb_ROI6[Task_version == "1"] - OxyHb_ROI6[Task_version == "2"])
shapiro.test(d6)
```

```
## 
##  Shapiro-Wilk normality test
## 
## data:  d6
## W = 0.98971, p-value = 0.9683
```

```
d7 <- with(myData2, OxyHb_ROI7[Task_version == "1"] - OxyHb_ROI7[Task_version == "2"])
shapiro.test(d7)
```

```
## 
##  Shapiro-Wilk normality test
## 
## data:  d7
## W = 0.97908, p-value = 0.6406
```

```
d8 <- with(myData2, OxyHb_ROI8[Task_version == "1"] - OxyHb_ROI8[Task_version == "2"])
shapiro.test(d8)
```

```
## 
##  Shapiro-Wilk normality test
## 
## data:  d8
## W = 0.98625, p-value = 0.8934
```

```
d9 <- with(myData2, OxyHb_ROI9[Task_version == "1"] - OxyHb_ROI9[Task_version == "2"])
shapiro.test(d9)
```

```
## 
##  Shapiro-Wilk normality test
## 
## data:  d9
## W = 0.98932, p-value = 0.9622
```

Perform t-tests for data with normal distribution. For data that is
not normally distributed, additional bootstrapping was performed. Effect
sizes were calculated for significant effects.

```
res_ROI1 <- t.test(OxyHb_ROI1 ~ Task_version, data = myData2, paired = TRUE)
res_ROI1
```

```
## 
##  Paired t-test
## 
## data:  OxyHb_ROI1 by Task_version
## t = -0.10301, df = 40, p-value = 0.9185
## alternative hypothesis: true mean difference is not equal to 0
## 95 percent confidence interval:
##  -0.02735733  0.02470385
## sample estimates:
## mean difference 
##    -0.001326741
```

```
res_ROI2 <- t.test(OxyHb_ROI2 ~ Task_version, data = myData2, paired = TRUE)
res_ROI2
```

```
## 
##  Paired t-test
## 
## data:  OxyHb_ROI2 by Task_version
## t = -0.85386, df = 40, p-value = 0.3983
## alternative hypothesis: true mean difference is not equal to 0
## 95 percent confidence interval:
##  -0.04892152  0.01986189
## sample estimates:
## mean difference 
##     -0.01452982
```

```
res_ROI3 <- t.test(OxyHb_ROI3 ~ Task_version, data = myData2, paired = TRUE)
res_ROI3
```

```
## 
##  Paired t-test
## 
## data:  OxyHb_ROI3 by Task_version
## t = 2.5083, df = 40, p-value = 0.01629
## alternative hypothesis: true mean difference is not equal to 0
## 95 percent confidence interval:
##  0.005904985 0.054894978
## sample estimates:
## mean difference 
##      0.03039998
```

```
myData2 %>% 
cohens_d(OxyHb_ROI3 ~ Task_version, paired = TRUE)
```

```
## # A tibble: 1 × 7
##   .y.        group1 group2 effsize    n1    n2 magnitude
## * <chr>      <chr>  <chr>    <dbl> <int> <int> <ord>    
## 1 OxyHb_ROI3 1      2        0.392    41    41 small
```

```
res_ROI4 <- t.test(OxyHb_ROI4 ~ Task_version, data = myData2, paired = TRUE)
res_ROI4
```

```
## 
##  Paired t-test
## 
## data:  OxyHb_ROI4 by Task_version
## t = 2.193, df = 40, p-value = 0.03418
## alternative hypothesis: true mean difference is not equal to 0
## 95 percent confidence interval:
##  0.002153838 0.052786259
## sample estimates:
## mean difference 
##      0.02747005
```

```
myData2 %>% 
cohens_d(OxyHb_ROI4 ~ Task_version, paired = TRUE)
```

```
## # A tibble: 1 × 7
##   .y.        group1 group2 effsize    n1    n2 magnitude
## * <chr>      <chr>  <chr>    <dbl> <int> <int> <ord>    
## 1 OxyHb_ROI4 1      2        0.342    41    41 small
```

```
# bootstrapping
sampling <- function(n, k){
  mySamples <- matrix(mapply(function(i) sample(1:n, n, replace = T), 1:k), ncol = n, nrow = k, byrow = T)
  return(mySamples)}

k <- 10000
n <- 41
ind <- sampling(n, k)

ci <- matrix(NA, ncol = 2, nrow = k)

for(i in 1:k) {ci[i, ] <- t.test(OxyHb_ROI4[c(ind[i, ], ind[i, ]+n)]~Task_version[c(ind[i, ], ind[i, ]+n)], data = myData2, paired = T, var.equal = T)$conf.int[1:2]}

myCI_OxyHb_ROI4 <- quantile(ci[1, ], probs = c(0.05, 0.95))
myCI_OxyHb_ROI4
```

```
##          5%         95% 
## 0.006143087 0.032467802
```

```
res_ROI5 <- t.test(OxyHb_ROI5 ~ Task_version, data = myData2, paired = TRUE)
res_ROI5
```

```
## 
##  Paired t-test
## 
## data:  OxyHb_ROI5 by Task_version
## t = 2.5246, df = 40, p-value = 0.01565
## alternative hypothesis: true mean difference is not equal to 0
## 95 percent confidence interval:
##  0.005002915 0.045167970
## sample estimates:
## mean difference 
##      0.02508544
```

```
myData2 %>% 
cohens_d(OxyHb_ROI5 ~ Task_version, paired = TRUE)
```

```
## # A tibble: 1 × 7
##   .y.        group1 group2 effsize    n1    n2 magnitude
## * <chr>      <chr>  <chr>    <dbl> <int> <int> <ord>    
## 1 OxyHb_ROI5 1      2        0.394    41    41 small
```

```
res_ROI6 <- t.test(OxyHb_ROI6 ~ Task_version, data = myData2, paired = TRUE)
res_ROI6
```

```
## 
##  Paired t-test
## 
## data:  OxyHb_ROI6 by Task_version
## t = 0.5139, df = 40, p-value = 0.6101
## alternative hypothesis: true mean difference is not equal to 0
## 95 percent confidence interval:
##  -0.01456256  0.02449335
## sample estimates:
## mean difference 
##     0.004965399
```

```
res_ROI7 <- t.test(OxyHb_ROI7 ~ Task_version, data = myData2, paired = TRUE)
res_ROI7
```

```
## 
##  Paired t-test
## 
## data:  OxyHb_ROI7 by Task_version
## t = 0.69836, df = 40, p-value = 0.489
## alternative hypothesis: true mean difference is not equal to 0
## 95 percent confidence interval:
##  -0.01652008  0.03396436
## sample estimates:
## mean difference 
##     0.008722139
```

```
res_ROI8 <- t.test(OxyHb_ROI8 ~ Task_version, data = myData2, paired = TRUE)
res_ROI8
```

```
## 
##  Paired t-test
## 
## data:  OxyHb_ROI8 by Task_version
## t = 2.1635, df = 40, p-value = 0.03653
## alternative hypothesis: true mean difference is not equal to 0
## 95 percent confidence interval:
##  0.001817442 0.053383796
## sample estimates:
## mean difference 
##      0.02760062
```

```
myData2 %>% 
cohens_d(OxyHb_ROI8 ~ Task_version, paired = TRUE)
```

```
## # A tibble: 1 × 7
##   .y.        group1 group2 effsize    n1    n2 magnitude
## * <chr>      <chr>  <chr>    <dbl> <int> <int> <ord>    
## 1 OxyHb_ROI8 1      2        0.338    41    41 small
```

```
res_ROI9 <- t.test(OxyHb_ROI9 ~ Task_version, data = myData2, paired = TRUE)
res_ROI9
```

```
## 
##  Paired t-test
## 
## data:  OxyHb_ROI9 by Task_version
## t = 1.3474, df = 40, p-value = 0.1854
## alternative hypothesis: true mean difference is not equal to 0
## 95 percent confidence interval:
##  -0.007789246  0.038947689
## sample estimates:
## mean difference 
##      0.01557922
```

## Prepare dataset for analyzing deoxy-Hb

```
myLongFormat2 <- myData[, c(1, 2, 23 )]

for(i in 1:1){
i <- i + 23
colnames(myData)[c(1, 2, i)] <- colnames(myLongFormat2)
myLongFormat2 <- rbind(myLongFormat2, myData[, c(1, 2, i)], make.row.names = F)
}

head(myLongFormat2)
```

```
##   Code Task_version   DeoxyHb_LH
## 1    1            1 -0.008670535
## 2    3            1 -0.044785009
## 3    4            1 -0.018917523
## 4    5            1 -0.021105805
## 5    6            1 -0.008031040
## 6    7            1  0.024014855
```

```
hemisphere <- as.numeric(gl(2, dim(myData)[1]))
myLongFormat2 <- cbind(myLongFormat2, as.numeric(substr(myLongFormat2[, 1], 1, 2)), hemisphere)
colnames(myLongFormat2)[c(4)] <- "pb"
colnames(myLongFormat2)[c(3)] <- "deoxy_Hb"

head(myLongFormat2)
```

```
##   Code Task_version     deoxy_Hb pb hemisphere
## 1    1            1 -0.008670535  1          1
## 2    3            1 -0.044785009  3          1
## 3    4            1 -0.018917523  4          1
## 4    5            1 -0.021105805  5          1
## 5    6            1 -0.008031040  6          1
## 6    7            1  0.024014855  7          1
```

## Linear mixed‐effects‐models (LMM) for deoxy-Hb

Now we calculate the linear mixed‐effects model for deoxy-Hb

```
m2  <- lmer(deoxy_Hb ~ Task_version*hemisphere +(1|pb), myLongFormat2, na.action = na.omit )
anova(m2, type = "I", ddf = "Satterthwaite")
```

```
## Type I Analysis of Variance Table with Satterthwaite's method
##                             Sum Sq    Mean Sq NumDF DenDF F value Pr(>F)
## Task_version            0.00002101 0.00002101     1   120  0.0801 0.7777
## hemisphere              0.00052779 0.00052779     1   120  2.0116 0.1587
## Task_version:hemisphere 0.00000032 0.00000032     1   120  0.0012 0.9720
```

Descriptive statistics: Means and SD of data

```
myLongFormat2 %>%
  group_by(Task_version, hemisphere) %>%
  get_summary_stats(deoxy_Hb, type = "mean_sd")
```

```
## # A tibble: 4 × 6
##   Task_version hemisphere variable     n   mean    sd
##          <int>      <dbl> <chr>    <dbl>  <dbl> <dbl>
## 1            1          1 deoxy_Hb    41 -0.011 0.025
## 2            1          2 deoxy_Hb    41 -0.015 0.024
## 3            2          1 deoxy_Hb    41 -0.012 0.022
## 4            2          2 deoxy_Hb    41 -0.015 0.02
```

```
myLongFormat2 %>%
  group_by(Task_version) %>%
  get_summary_stats(deoxy_Hb, type = "mean_sd")
```

```
## # A tibble: 2 × 5
##   Task_version variable     n   mean    sd
##          <int> <chr>    <dbl>  <dbl> <dbl>
## 1            1 deoxy_Hb    82 -0.013 0.024
## 2            2 deoxy_Hb    82 -0.014 0.021
```

```
myLongFormat2 %>%
  group_by(hemisphere) %>%
  get_summary_stats(deoxy_Hb, type = "mean_sd")
```

```
## # A tibble: 2 × 5
##   hemisphere variable     n   mean    sd
##        <dbl> <chr>    <dbl>  <dbl> <dbl>
## 1          1 deoxy_Hb    82 -0.011 0.023
## 2          2 deoxy_Hb    82 -0.015 0.022
```

Checking assumptions

```
residuals_m2 <- resid(m2)
fitted_m2 <- fitted(m2)

plot(residuals_m2[1:41] ~ fitted_m2[1:41], main = "Residuals - Game, left hemisphere")
```

```
qqnorm(residuals_m2[1:41], main = "QQ-Plot - Game, left hemisphere")
qqline(residuals_m2[1:41])
```

```
jarque.test(residuals_m2[1:41])
```

```
## 
##  Jarque-Bera Normality Test
## 
## data:  residuals_m2[1:41]
## JB = 3.3835, p-value = 0.1842
## alternative hypothesis: greater
```

```
plot(residuals_m2[42:82] ~ fitted_m2[42:82], main = "Residuals - Non-Game, left hemisphere")
```

```
qqnorm(residuals_m2[42:82], main = "QQ-Plot - Non-Game, left hemisphere")
qqline(residuals_m2[42:82])
```

```
jarque.test(residuals_m2[42:82])
```

```
## 
##  Jarque-Bera Normality Test
## 
## data:  residuals_m2[42:82]
## JB = 16.527, p-value = 0.0002578
## alternative hypothesis: greater
```

```
plot(residuals_m2[83:123] ~ fitted_m2[83:123], main = "Residuals - Game, right hemisphere")
```

```
qqnorm(residuals_m2[83:123], main = "QQ-Plot - Game, right hemisphere")
qqline(residuals_m2[83:123])
```

```
jarque.test(residuals_m2[83:123])
```

```
## 
##  Jarque-Bera Normality Test
## 
## data:  residuals_m2[83:123]
## JB = 0.88933, p-value = 0.641
## alternative hypothesis: greater
```

```
plot(residuals_m2[124:164] ~ fitted_m2[124:164], main = "Residuals - Non-Game, right hemisphere")
```

```
qqnorm(residuals_m2[124:164], main = "QQ-Plot - Non-Game, right hemisphere")
qqline(residuals_m2[124:164])
```

```
jarque.test(residuals_m2[124:164])
```

```
## 
##  Jarque-Bera Normality Test
## 
## data:  residuals_m2[124:164]
## JB = 0.5903, p-value = 0.7444
## alternative hypothesis: greater
```

According to the Jarque-Bera Normality Test, skewness and kurtosis of
the data match a normal distribution except for the non-game condition -
left hemisphere. Therefore, in the next step we check the data for
possible outliers.

Checking for outliers using Box-Plots

```
ggplot(myLongFormat2, aes(factor(Task_version), deoxy_Hb, fill = factor(hemisphere))) + geom_boxplot() +
    scale_fill_brewer(palette = "Dark2",
    name = "Hemisphere",
    labels =    c("left", "right")) +
    theme_classic() +
    scale_x_discrete(name = "Game condition",
    labels = c("Game", "Non-Game")) +
    scale_y_continuous(name = "Deoxy Hb [micromol]")
```

We see that there are potential outliers (values larger or smaller
1.5 times the IQR) in the game and non-game condition. Let’s exclude
these outlier values and recalculate the mixed-effects model to see if
results would change.

```
Game_LH_deoxy <- subset(myLongFormat2[myLongFormat2$Task_version == 1 & myLongFormat2$hemisphere == 1, ], )

# create a vector of outliers for the numeric factor
outliers_Game_LH_deoxy <- boxplot(Game_LH_deoxy$deoxy_Hb, plot = FALSE)$out

# Replace the values with NA
Game_LH_deoxy[Game_LH_deoxy$deoxy_Hb %in% outliers_Game_LH_deoxy, "deoxy_Hb"] = NA

Game_RH_deoxy <- subset(myLongFormat2[myLongFormat2$Task_version == 1 & myLongFormat2$hemisphere == 2, ], )

# create a vector of outliers for the numeric factor
outliers_Game_RH_deoxy <- boxplot(Game_RH_deoxy$deoxy_Hb, plot = FALSE)$out

# Replace the values with NA
Game_RH_deoxy[Game_RH_deoxy$deoxy_Hb %in% outliers_Game_RH_deoxy, "deoxy_Hb"] = NA

NoGame_LH_deoxy <- subset(myLongFormat2[myLongFormat2$Task_version == 2 & myLongFormat2$hemisphere == 1, ], )

# create a vector of outliers for the numeric factor
outliers_NoGame_LH_deoxy <- boxplot(NoGame_LH_deoxy$deoxy_Hb, plot = FALSE)$out

# Replace the values with NA
NoGame_LH_deoxy[NoGame_LH_deoxy$deoxy_Hb %in% outliers_NoGame_LH_deoxy, "deoxy_Hb"] = NA

NoGame_RH_deoxy <- subset(myLongFormat2[myLongFormat2$Task_version == 2 & myLongFormat2$hemisphere == 2, ], )

# create a vector of outliers for the numeric factor
outliers_NoGame_RH_deoxy <- boxplot(NoGame_RH_deoxy$deoxy_Hb, plot = FALSE)$out

# Replace the values with NA
NoGame_RH_deoxy[NoGame_RH_deoxy$deoxy_Hb %in% outliers_NoGame_RH_deoxy, "deoxy_Hb"] = NA

myLongFormat2_noOutliers <- rbind(Game_LH_deoxy, Game_RH_deoxy, NoGame_LH_deoxy, NoGame_RH_deoxy)

m2_noOutliers  <- lmer(deoxy_Hb ~ Task_version*hemisphere +(1|pb), myLongFormat2_noOutliers, na.action = na.omit )
anova(m2_noOutliers, type = "I", ddf = "Satterthwaite")
```

```
## Type I Analysis of Variance Table with Satterthwaite's method
##                             Sum Sq    Mean Sq NumDF  DenDF F value Pr(>F)
## Task_version            0.00006017 0.00006017     1 113.61  0.2845 0.5948
## hemisphere              0.00044215 0.00044215     1 112.74  2.0905 0.1510
## Task_version:hemisphere 0.00010600 0.00010600     1 112.81  0.5012 0.4804
```

Results are the same with and without exclusion of outliers.
Therefore, we decided to report on the analysis of the whole data set in
the manuscript.

## T-Tests per ROI for deoxy-Hb

Plot mean and SE per ROI and task condition.

```
ROI1_deoxy_mean <- ggerrorplot(myData2, x = "Task_version", y = "DeoxyHb_ROI1", 
            desc_stat = "mean_se",
            ylim = c(-0.04, 0.005),
            error.plot = "errorbar",            # Change error plot type
            add = "mean"                        # Add mean points
            )

ROI2_deoxy_mean <- ggerrorplot(myData2, x = "Task_version", y = "DeoxyHb_ROI2", 
            desc_stat = "mean_se",
            error.plot = "errorbar",            # Change error plot type
            ylim = c(-0.04, 0.005),
                add = "mean"                        # Add mean points
            )

ROI3_deoxy_mean <- ggerrorplot(myData2, x = "Task_version", y = "DeoxyHb_ROI3", 
            desc_stat = "mean_se",
            error.plot = "errorbar",            # Change error plot type
            ylim = c(-0.04, 0.005),
                add = "mean"                        # Add mean points
            )

ROI4_deoxy_mean <- ggerrorplot(myData2, x = "Task_version", y = "DeoxyHb_ROI4", 
            desc_stat = "mean_se",
            error.plot = "errorbar",            # Change error plot type
            ylim = c(-0.04, 0.005),
                add = "mean"                        # Add mean points
            )


ROI5_deoxy_mean <- ggerrorplot(myData2, x = "Task_version", y = "DeoxyHb_ROI5", 
            desc_stat = "mean_se",
            error.plot = "errorbar",            # Change error plot type
            ylim = c(-0.04, 0.005),
                add = "mean"                        # Add mean points
            )

ROI6_deoxy_mean <- ggerrorplot(myData2, x = "Task_version", y = "DeoxyHb_ROI6", 
            desc_stat = "mean_se",
            error.plot = "errorbar",            # Change error plot type
            ylim = c(-0.04, 0.005),
                add = "mean"                        # Add mean points
            )

ROI7_deoxy_mean <- ggerrorplot(myData2, x = "Task_version", y = "DeoxyHb_ROI7", 
            desc_stat = "mean_se",
            error.plot = "errorbar",            # Change error plot type
            ylim = c(-0.04, 0.005),
                add = "mean"                        # Add mean points
            )

ROI8_deoxy_mean <- ggerrorplot(myData2, x = "Task_version", y = "DeoxyHb_ROI8", 
            desc_stat = "mean_se",
            error.plot = "errorbar",            # Change error plot type
            ylim = c(-0.04, 0.005),
                add = "mean"                        # Add mean points
            )

ROI9_deoxy_mean <- ggerrorplot(myData2, x = "Task_version", y = "DeoxyHb_ROI9", 
            desc_stat = "mean_se",
            error.plot = "errorbar",            # Change error plot type
            ylim = c(-0.04, 0.005),
                add = "mean"                        # Add mean points
            )


ggarrange(ROI1_deoxy_mean, ROI2_deoxy_mean, ROI3_deoxy_mean, ROI4_deoxy_mean, ROI5_deoxy_mean, ROI6_deoxy_mean,
        ROI7_deoxy_mean, ROI8_deoxy_mean, ROI9_deoxy_mean,
         labels = c("ROI1", "ROI2", "ROI3", "ROI4", "ROI5", "ROI6", "ROI7", "ROI8", "ROI9"),
           ncol = 3, nrow = 3)
```

Check normal distribution.

```
# compute the difference
d1_deoxy <- with(myData2, DeoxyHb_ROI1[Task_version == "1"] - DeoxyHb_ROI1[Task_version == "2"])
# Shapiro-Wilk normality test for the differences
shapiro.test(d1_deoxy)
```

```
## 
##  Shapiro-Wilk normality test
## 
## data:  d1_deoxy
## W = 0.96124, p-value = 0.1739
```

```
d2_deoxy <- with(myData2, DeoxyHb_ROI2[Task_version == "1"] - DeoxyHb_ROI2[Task_version == "2"])
shapiro.test(d2_deoxy)
```

```
## 
##  Shapiro-Wilk normality test
## 
## data:  d2_deoxy
## W = 0.97645, p-value = 0.5442
```

```
d3_deoxy <- with(myData2, DeoxyHb_ROI3[Task_version == "1"] - DeoxyHb_ROI3[Task_version == "2"])
shapiro.test(d3_deoxy)
```

```
## 
##  Shapiro-Wilk normality test
## 
## data:  d3_deoxy
## W = 0.87824, p-value = 0.0004014
```

```
d4_deoxy <- with(myData2, DeoxyHb_ROI4[Task_version == "1"] - DeoxyHb_ROI4[Task_version == "2"])
shapiro.test(d4_deoxy)
```

```
## 
##  Shapiro-Wilk normality test
## 
## data:  d4_deoxy
## W = 0.9722, p-value = 0.4058
```

```
d5_deoxy <- with(myData2, DeoxyHb_ROI5[Task_version == "1"] - DeoxyHb_ROI5[Task_version == "2"])
shapiro.test(d5_deoxy)
```

```
## 
##  Shapiro-Wilk normality test
## 
## data:  d5_deoxy
## W = 0.91975, p-value = 0.006699
```

```
d6_deoxy <- with(myData2, DeoxyHb_ROI6[Task_version == "1"] - DeoxyHb_ROI6[Task_version == "2"])
shapiro.test(d6_deoxy)
```

```
## 
##  Shapiro-Wilk normality test
## 
## data:  d6_deoxy
## W = 0.93178, p-value = 0.01655
```

```
d7_deoxy <- with(myData2, DeoxyHb_ROI7[Task_version == "1"] - DeoxyHb_ROI7[Task_version == "2"])
shapiro.test(d7_deoxy)
```

```
## 
##  Shapiro-Wilk normality test
## 
## data:  d7_deoxy
## W = 0.96853, p-value = 0.3086
```

```
d8_deoxy <- with(myData2, DeoxyHb_ROI8[Task_version == "1"] - DeoxyHb_ROI8[Task_version == "2"])
shapiro.test(d8_deoxy)
```

```
## 
##  Shapiro-Wilk normality test
## 
## data:  d8_deoxy
## W = 0.94559, p-value = 0.04904
```

```
d9_deoxy <- with(myData2, DeoxyHb_ROI9[Task_version == "1"] - DeoxyHb_ROI9[Task_version == "2"])
shapiro.test(d9_deoxy)
```

```
## 
##  Shapiro-Wilk normality test
## 
## data:  d9_deoxy
## W = 0.95386, p-value = 0.09566
```

Perform t-tests for data with normal distribution. For data that is
not normally distributed, additional bootstrapping was performed. Effect
sizes were calculated for significant effects.

```
res_ROI1_deoxy <- t.test(DeoxyHb_ROI1 ~ Task_version, data = myData2, paired = TRUE)
res_ROI1_deoxy
```

```
## 
##  Paired t-test
## 
## data:  DeoxyHb_ROI1 by Task_version
## t = -0.38915, df = 40, p-value = 0.6992
## alternative hypothesis: true mean difference is not equal to 0
## 95 percent confidence interval:
##  -0.014264484  0.009658209
## sample estimates:
## mean difference 
##    -0.002303137
```

```
res_ROI2_deoxy <- t.test(DeoxyHb_ROI2 ~ Task_version, data = myData2, paired = TRUE)
res_ROI2_deoxy
```

```
## 
##  Paired t-test
## 
## data:  DeoxyHb_ROI2 by Task_version
## t = -0.14327, df = 40, p-value = 0.8868
## alternative hypothesis: true mean difference is not equal to 0
## 95 percent confidence interval:
##  -0.01321369  0.01146432
## sample estimates:
## mean difference 
##   -0.0008746854
```

```
res_ROI3_deoxy <- t.test(DeoxyHb_ROI3 ~ Task_version, data = myData2, paired = TRUE)
res_ROI3_deoxy
```

```
## 
##  Paired t-test
## 
## data:  DeoxyHb_ROI3 by Task_version
## t = 1.3969, df = 40, p-value = 0.1701
## alternative hypothesis: true mean difference is not equal to 0
## 95 percent confidence interval:
##  -0.003826401  0.020954468
## sample estimates:
## mean difference 
##     0.008564034
```

```
# bootstrapping
for(i in 1:k) {ci[i, ] <- t.test(DeoxyHb_ROI3[c(ind[i, ], ind[i, ]+n)]~Task_version[c(ind[i, ], ind[i, ]+n)], data = myData2, paired = T, var.equal = T)$conf.int[1:2]}

myCI_DeoxyHb_ROI3 <- quantile(ci[1, ], probs = c(0.05, 0.95))
myCI_DeoxyHb_ROI3
```

```
##           5%          95% 
## -0.005632134  0.010458385
```

```
res_ROI4_deoxy <- t.test(DeoxyHb_ROI4 ~ Task_version, data = myData2, paired = TRUE)
res_ROI4_deoxy
```

```
## 
##  Paired t-test
## 
## data:  DeoxyHb_ROI4 by Task_version
## t = 0.21158, df = 40, p-value = 0.8335
## alternative hypothesis: true mean difference is not equal to 0
## 95 percent confidence interval:
##  -0.009748139  0.012027744
## sample estimates:
## mean difference 
##     0.001139803
```

```
res_ROI5_deoxy <- t.test(DeoxyHb_ROI5 ~ Task_version, data = myData2, paired = TRUE)
res_ROI5_deoxy
```

```
## 
##  Paired t-test
## 
## data:  DeoxyHb_ROI5 by Task_version
## t = -1.7664, df = 40, p-value = 0.08497
## alternative hypothesis: true mean difference is not equal to 0
## 95 percent confidence interval:
##  -0.018869801  0.001269049
## sample estimates:
## mean difference 
##    -0.008800376
```

```
# bootstrapping
for(i in 1:k) {ci[i, ] <- t.test(DeoxyHb_ROI5[c(ind[i, ], ind[i, ]+n)]~Task_version[c(ind[i, ], ind[i, ]+n)], data = myData2, paired = T, var.equal = T)$conf.int[1:2]}

myCI_DeoxyHb_ROI5 <- quantile(ci[1, ], probs = c(0.05, 0.95))
myCI_DeoxyHb_ROI5
```

```
##           5%          95% 
## -0.016045574 -0.000663502
```

```
res_ROI6_deoxy <- t.test(DeoxyHb_ROI6 ~ Task_version, data = myData2, paired = TRUE)
res_ROI6_deoxy
```

```
## 
##  Paired t-test
## 
## data:  DeoxyHb_ROI6 by Task_version
## t = -0.70771, df = 40, p-value = 0.4832
## alternative hypothesis: true mean difference is not equal to 0
## 95 percent confidence interval:
##  -0.01323194  0.00636851
## sample estimates:
## mean difference 
##    -0.003431716
```

```
# bootstrapping
for(i in 1:k) {ci[i, ] <- t.test(DeoxyHb_ROI6[c(ind[i, ], ind[i, ]+n)]~Task_version[c(ind[i, ], ind[i, ]+n)], data = myData2, paired = T, var.equal = T)$conf.int[1:2]}

myCI_DeoxyHb_ROI6 <- quantile(ci[1, ], probs = c(0.05, 0.95))
myCI_DeoxyHb_ROI6
```

```
##           5%          95% 
## -0.004691037  0.008105657
```

```
res_ROI7_deoxy <- t.test(DeoxyHb_ROI7 ~ Task_version, data = myData2, paired = TRUE)
res_ROI7_deoxy
```

```
## 
##  Paired t-test
## 
## data:  DeoxyHb_ROI7 by Task_version
## t = 1.589, df = 40, p-value = 0.1199
## alternative hypothesis: true mean difference is not equal to 0
## 95 percent confidence interval:
##  -0.002128474  0.017784566
## sample estimates:
## mean difference 
##     0.007828046
```

```
res_ROI8_deoxy <- t.test(DeoxyHb_ROI8 ~ Task_version, data = myData2, paired = TRUE)
res_ROI8_deoxy
```

```
## 
##  Paired t-test
## 
## data:  DeoxyHb_ROI8 by Task_version
## t = -0.57104, df = 40, p-value = 0.5712
## alternative hypothesis: true mean difference is not equal to 0
## 95 percent confidence interval:
##  -0.013061085  0.007306437
## sample estimates:
## mean difference 
##    -0.002877324
```

```
# bootstrapping
for(i in 1:k) {ci[i, ] <- t.test(DeoxyHb_ROI8[c(ind[i, ], ind[i, ]+n)]~Task_version[c(ind[i, ], ind[i, ]+n)], data = myData2, paired = T, var.equal = T)$conf.int[1:2]}

myCI_DeoxyHb_ROI8 <- quantile(ci[1, ], probs = c(0.05, 0.95))
myCI_DeoxyHb_ROI8
```

```
##           5%          95% 
## -0.007806211  0.010216497
```

```
res_ROI9_deoxy <- t.test(DeoxyHb_ROI9 ~ Task_version, data = myData2, paired = TRUE)
res_ROI9_deoxy
```

```
## 
##  Paired t-test
## 
## data:  DeoxyHb_ROI9 by Task_version
## t = 0.43082, df = 40, p-value = 0.6689
## alternative hypothesis: true mean difference is not equal to 0
## 95 percent confidence interval:
##  -0.008738026  0.013472561
## sample estimates:
## mean difference 
##     0.002367268
```
