# Supplementary material for: Engaging learners with games–Insights from functional near-infrared spectroscopy
Source: PLoS One. 2023 Jun 6;18(6):e0286450. doi: 10.1371/journal.pone.0286450 (PMC10243642; doi:10.1371/journal.pone.0286450)
Supplement: S2 File — RMarkdown script for the analysis of the behavioral data, i.e., task performance, user experience, etc. (HTML) [file pone.0286450.s002.html]

Supplementary Material S2: R Code for statistical analysis of task performance and user experience


# Supplementary Material S2: R Code for statistical analysis of task performance and user experience

#### 2022-12-12

This RMarkdown shows the analysis of the task performance and user
experience using t-tests in R (version 4.2.1).

## Loading R packages and importing dataset

```
library(ggplot2) # to plot data
library(tidyverse) # for cleaning up data / plotting / descriptive statistic
library(PairedData) # for paired data analysis
library(stats) # for statistical analyses
library(ggpubr) # to plot data
library(rstatix) # for calculating effect sizes t-tests
library(coin) # for calculating effect sizes Wilcoxon tests
```

```
# set working directory, where the S4_File.txt file is stored
setwd("E:/")
myData <- read.table("S4_File.txt", header = T, dec = ".")
head(myData)
```

```
##   Code Gender Age Task_version MDBF_GS MDBF_WM MDBF_RU FKS_glatt FKS_absorb
## 1    1      1  23            2      40      27      37        36         14
## 2    3      1  25            2      36      30      38        32         13
## 3    4      1  18            2      34      26      36        32         14
## 4    5      1  21            2      25      31      32        39         18
## 5    6      2  20            2      33      16      33        32         12
## 6    7      1  23            2      34      23      29        34         16
##   FKS_ges FKS_besorg FKS_pass UEQ_Att UEQ_Pers UEQ_Eff UEQ_Dep UEQ_Stim UEQ_Nov
## 1      50          5       10   1.500     1.25    0.75    0.50     0.50    1.25
## 2      45          3       13   0.167    -2.25    0.00    0.00     0.00    0.25
## 3      46          5       13   0.667     3.00    1.00    1.25    -0.75   -1.00
## 4      57         12       12   1.333     2.75    0.75    2.00     1.00   -0.75
## 5      44          7       15   0.333     2.50    1.75    0.50    -0.50   -1.50
## 6      50          6       11  -0.833     2.75    1.50    1.00    -0.50   -0.25
##    accuracy    errors       IHR      HRV
## 1 0.9483148  90.90909        NA       NA
## 2 0.9553989  91.66667  89.98904 41.73693
## 3 0.9699656 100.00000 101.34221 53.52283
## 4 0.9597171  93.18182  75.94167 59.76344
## 5 0.9459332  88.63636  79.53779 62.09524
## 6 0.9610541  95.45455  72.32449 93.57713
```

```
# Code: Participant code
# Gender: 1 = Female, 2 = Male
# Task_version: 1 = Game-based task version, 2 = non-game-based task version
# MDBF_GS: Mehrdimensionaler Befindlichkeitsfragebogen (English version: Multidimensional Mood State Questionnaire) - subscale "Good-bad mood"
# MDBF_WM: Mehrdimensionaler Befindlichkeitsfragebogen (English version: Multidimensional Mood State Questionnaire) - subscale "Awake-tired"
# MDBF_RU: Mehrdimensionaler Befindlichkeitsfragebogen (English version: Multidimensional Mood State Questionnaire) - subscale "Calm-nervous"
# FKS_glatt: Flow Short Scale - subscale "Fluency"
# FKS_absorb: FLow Short Scale - subscale "Absorption"
# FKS_ges: Flow Short Scale - subscale "Geenral factor"
# FKS_besorg: Flow Short Scale - subscale "Concern"
# FKS_pass: Flow Short Scale - subscale "Perceived fit of demands and skills"
# UEQ_Att: User Experience Questionnaire - subscale "Attractiveness"
# UEQ_Pers: User Experience Questionnaire - subscale "Perspicuity"
# UEQ_Eff: User Experience Questionnaire - subscale "Efficiency"
# UEQ_Dep: User Experience Questionnaire - subscale "Dependability"
# UEQ_Stim: User Experience Questionnaire - subscale "Stimulation"
# UEQ_Nov: User Experience Questionnaire - subscale "Novelty"
# accuracy: Task performance - participants’ guesses’ mean accuracy across all fraction estimation items 
# errors: Task performance - participants’ hit rate
# IHR: heart rate
# HRV: heart rate variability
```

Descriptive statistics: Age for male (2) and female (1)
participants

```
myData2 <- myData[myData[,"Task_version"]=="1",]

group_by(myData2, Gender) %>%
  summarise(
    count = n(),
    mean_Age = mean(Age, na.rm = TRUE),
    sd_Age = sd(Age, na.rm = TRUE)
    )
```

```
## # A tibble: 2 × 4
##   Gender count mean_Age sd_Age
##    <int> <int>    <dbl>  <dbl>
## 1      1    20     22.4   2.62
## 2      2    21     23.5   3.37
```

## Analyzing task performance

Descriptive statistics: Means and SDs.

```
group_by(myData, Task_version) %>%
  summarise(
    count = n(),
    mean_accuracy = mean(accuracy, na.rm = TRUE),
    sd_accuracy = sd(accuracy, na.rm = TRUE)
    )
```

```
## # A tibble: 2 × 4
##   Task_version count mean_accuracy sd_accuracy
##          <int> <int>         <dbl>       <dbl>
## 1            1    41         0.956     0.00891
## 2            2    41         0.960     0.00924
```

```
group_by(myData, Task_version) %>%
  summarise(
    count = n(),
    mean_correct_answ = mean(errors, na.rm = TRUE),
    sd_correct_answ = sd(errors, na.rm = TRUE)
    )
```

```
## # A tibble: 2 × 4
##   Task_version count mean_correct_answ sd_correct_answ
##          <int> <int>             <dbl>           <dbl>
## 1            1    41              93.3            4.47
## 2            2    41              94.0            4.32
```

Check normal distribution.

```
# compute the difference
d_accuracy <- with(myData, accuracy[Task_version == "1"] - accuracy[Task_version == "2"])
d_errors <- with(myData, errors[Task_version == "1"] - errors[Task_version == "2"])

# Shapiro-Wilk normality test for the differences
shapiro.test(d_accuracy)
```

```
## 
##  Shapiro-Wilk normality test
## 
## data:  d_accuracy
## W = 0.97097, p-value = 0.3707
```

```
shapiro.test(d_errors)
```

```
## 
##  Shapiro-Wilk normality test
## 
## data:  d_errors
## W = 0.96371, p-value = 0.2117
```

Perform t-tests for data with normal distribution. Effect sizes were
calculated for significant effects.

```
ttest_accuracy <- t.test(accuracy ~ Task_version, data = myData, paired = TRUE)
ttest_accuracy
```

```
## 
##  Paired t-test
## 
## data:  accuracy by Task_version
## t = -1.9072, df = 40, p-value = 0.0637
## alternative hypothesis: true mean difference is not equal to 0
## 95 percent confidence interval:
##  -0.0073643008  0.0002135491
## sample estimates:
## mean difference 
##    -0.003575376
```

```
ttest_errors <- t.test(errors ~ Task_version, data = myData, paired = TRUE)
ttest_errors
```

```
## 
##  Paired t-test
## 
## data:  errors by Task_version
## t = -0.76271, df = 40, p-value = 0.4501
## alternative hypothesis: true mean difference is not equal to 0
## 95 percent confidence interval:
##  -2.464835  1.114187
## sample estimates:
## mean difference 
##      -0.6753239
```

## Analyzing the Multidimensional Mood State Questionnaire (MDBF)

Descriptive statistics: Means and SDs.

```
group_by(myData, Task_version) %>%
  summarise(
    count = n(),
    mean_MDBF_GS = mean(MDBF_GS, na.rm = TRUE),
    sd_MDBF_GS = sd(MDBF_GS, na.rm = TRUE)
    )
```

```
## # A tibble: 2 × 4
##   Task_version count mean_MDBF_GS sd_MDBF_GS
##          <int> <int>        <dbl>      <dbl>
## 1            1    41         32.8       4.23
## 2            2    41         32.8       4.73
```

```
group_by(myData, Task_version) %>%
  summarise(
    count = n(),
    mean_MDBF_WM = mean(MDBF_WM, na.rm = TRUE),
    sd_MDBF_WM = sd(MDBF_WM, na.rm = TRUE)
    )
```

```
## # A tibble: 2 × 4
##   Task_version count mean_MDBF_WM sd_MDBF_WM
##          <int> <int>        <dbl>      <dbl>
## 1            1    41         25.9       6.93
## 2            2    41         25.0       6.52
```

```
group_by(myData, Task_version) %>%
  summarise(
    count = n(),
    mean_MDBF_RU = mean(MDBF_RU, na.rm = TRUE),
    sd_MDBF_RU = sd(MDBF_RU, na.rm = TRUE)
    )
```

```
## # A tibble: 2 × 4
##   Task_version count mean_MDBF_RU sd_MDBF_RU
##          <int> <int>        <dbl>      <dbl>
## 1            1    41         32.2       5.33
## 2            2    41         32.7       4.46
```

Check normal distribution.

```
# compute the difference
d_MDBF_GS <- with(myData, MDBF_GS[Task_version == "1"] - MDBF_GS[Task_version == "2"])
d_MDBF_WM <- with(myData, MDBF_WM[Task_version == "1"] - MDBF_WM[Task_version == "2"])
d_MDBF_RU <- with(myData, MDBF_RU[Task_version == "1"] - MDBF_RU[Task_version == "2"])

# Shapiro-Wilk normality test for the differences
shapiro.test(d_MDBF_GS)
```

```
## 
##  Shapiro-Wilk normality test
## 
## data:  d_MDBF_GS
## W = 0.94117, p-value = 0.03447
```

```
shapiro.test(d_MDBF_WM)
```

```
## 
##  Shapiro-Wilk normality test
## 
## data:  d_MDBF_WM
## W = 0.96758, p-value = 0.2869
```

```
shapiro.test(d_MDBF_RU)
```

```
## 
##  Shapiro-Wilk normality test
## 
## data:  d_MDBF_RU
## W = 0.95392, p-value = 0.09616
```

Perform t-tests for data with normal distribution. For data that is
not normally distributed, additional bootstrapping was performed. Effect
sizes were calculated for significant effects.

```
ttest_MDBF_GS <- t.test(MDBF_GS ~ Task_version, data = myData, paired = TRUE)
ttest_MDBF_GS
```

```
## 
##  Paired t-test
## 
## data:  MDBF_GS by Task_version
## t = 0, df = 40, p-value = 1
## alternative hypothesis: true mean difference is not equal to 0
## 95 percent confidence interval:
##  -1.131468  1.131468
## sample estimates:
## mean difference 
##               0
```

```
# bootstrapping
sampling <- function(n, k){
  mySamples <- matrix(mapply(function(i) sample(1:n, n, replace = T), 1:k), ncol = n, nrow = k, byrow = T)
  return(mySamples)}

k <- 10000
n <- 41
ind <- sampling(n, k)

ci <- matrix(NA, ncol = 2, nrow = k)

for(i in 1:k) {ci[i, ] <- t.test(MDBF_GS[c(ind[i, ], ind[i, ]+n)]~Task_version[c(ind[i, ], ind[i, ]+n)], data = myData, paired = T, var.equal = T)$conf.int[1:2]}

myCI_MDBF_GS <- quantile(ci[1, ], probs = c(0.05, 0.95))
myCI_MDBF_GS
```

```
##         5%        95% 
## -1.1463559  0.5122096
```

```
ttest_MDBF_WM <- t.test(MDBF_WM ~ Task_version, data = myData, paired = TRUE)
ttest_MDBF_WM
```

```
## 
##  Paired t-test
## 
## data:  MDBF_WM by Task_version
## t = 1.1041, df = 40, p-value = 0.2761
## alternative hypothesis: true mean difference is not equal to 0
## 95 percent confidence interval:
##  -0.7494792  2.5543572
## sample estimates:
## mean difference 
##        0.902439
```

```
ttest_MDBF_RU <- t.test(MDBF_RU ~ Task_version, data = myData, paired = TRUE)
ttest_MDBF_RU
```

```
## 
##  Paired t-test
## 
## data:  MDBF_RU by Task_version
## t = -0.8012, df = 40, p-value = 0.4277
## alternative hypothesis: true mean difference is not equal to 0
## 95 percent confidence interval:
##  -1.8042383  0.7798481
## sample estimates:
## mean difference 
##      -0.5121951
```

## Analyzing Flow experience (FKS)

Descriptive statistics: Means and SDs.

```
group_by(myData, Task_version) %>%
  summarise(
    count = n(),
    mean_FKS_glatt = mean(FKS_glatt, na.rm = TRUE),
    sd_FKS_glatt = sd(FKS_glatt, na.rm = TRUE)
    )
```

```
## # A tibble: 2 × 4
##   Task_version count mean_FKS_glatt sd_FKS_glatt
##          <int> <int>          <dbl>        <dbl>
## 1            1    41           31.9         5.76
## 2            2    41           30.6         6.10
```

```
group_by(myData, Task_version) %>%
  summarise(
    count = n(),
    mean_FKS_absorb = mean(FKS_absorb, na.rm = TRUE),
    sd_FKS_absorb = sd(FKS_absorb, na.rm = TRUE)
    )
```

```
## # A tibble: 2 × 4
##   Task_version count mean_FKS_absorb sd_FKS_absorb
##          <int> <int>           <dbl>         <dbl>
## 1            1    41            14.7          4.37
## 2            2    41            13.4          3.42
```

```
group_by(myData, Task_version) %>%
  summarise(
    count = n(),
    mean_FKS_ges = mean(FKS_ges, na.rm = TRUE),
    sd_FKS_ges = sd(FKS_ges, na.rm = TRUE)
    )
```

```
## # A tibble: 2 × 4
##   Task_version count mean_FKS_ges sd_FKS_ges
##          <int> <int>        <dbl>      <dbl>
## 1            1    41         46.5       8.18
## 2            2    41         44.0       7.72
```

```
group_by(myData, Task_version) %>%
  summarise(
    count = n(),
    mean_FKS_besorg = mean(FKS_besorg, na.rm = TRUE),
    sd_FKS_besorg = sd(FKS_besorg, na.rm = TRUE)
    )
```

```
## # A tibble: 2 × 4
##   Task_version count mean_FKS_besorg sd_FKS_besorg
##          <int> <int>           <dbl>         <dbl>
## 1            1    41            5.73          3.48
## 2            2    41            5.95          3.26
```

```
group_by(myData, Task_version) %>%
  summarise(
    count = n(),
    mean_FKS_pass = mean(FKS_pass, na.rm = TRUE),
    sd_FKS_pass = sd(FKS_pass, na.rm = TRUE)
    )
```

```
## # A tibble: 2 × 4
##   Task_version count mean_FKS_pass sd_FKS_pass
##          <int> <int>         <dbl>       <dbl>
## 1            1    41          12.1        1.95
## 2            2    41          12.2        2.11
```

Check normal distribution.

```
# compute the difference
d_FKS_glatt <- with(myData, FKS_glatt[Task_version == "1"] - FKS_glatt[Task_version == "2"])
d_FKS_absorb <- with(myData, FKS_absorb[Task_version == "1"] - FKS_absorb[Task_version == "2"])
d_FKS_ges <- with(myData, FKS_ges[Task_version == "1"] - FKS_ges[Task_version == "2"])
d_FKS_besorg <- with(myData, FKS_besorg[Task_version == "1"] - FKS_besorg[Task_version == "2"])
d_FKS_pass <- with(myData, FKS_pass[Task_version == "1"] - FKS_pass[Task_version == "2"])

# Shapiro-Wilk normality test for the differences
shapiro.test(d_FKS_glatt)
```

```
## 
##  Shapiro-Wilk normality test
## 
## data:  d_FKS_glatt
## W = 0.92241, p-value = 0.008151
```

```
shapiro.test(d_FKS_absorb)
```

```
## 
##  Shapiro-Wilk normality test
## 
## data:  d_FKS_absorb
## W = 0.97986, p-value = 0.67
```

```
shapiro.test(d_FKS_ges)
```

```
## 
##  Shapiro-Wilk normality test
## 
## data:  d_FKS_ges
## W = 0.97723, p-value = 0.5722
```

```
shapiro.test(d_FKS_besorg)
```

```
## 
##  Shapiro-Wilk normality test
## 
## data:  d_FKS_besorg
## W = 0.92277, p-value = 0.008376
```

```
shapiro.test(d_FKS_pass)
```

```
## 
##  Shapiro-Wilk normality test
## 
## data:  d_FKS_pass
## W = 0.94854, p-value = 0.06219
```

Perform t-tests for data with normal distribution. For data that is
not normally distributed, additional bootstrapping was performed. Effect
sizes were calculated for significant effects.

```
ttest_FKS_glatt <- t.test(FKS_glatt ~ Task_version, data = myData, paired = TRUE)
ttest_FKS_glatt
```

```
## 
##  Paired t-test
## 
## data:  FKS_glatt by Task_version
## t = 1.2576, df = 40, p-value = 0.2158
## alternative hypothesis: true mean difference is not equal to 0
## 95 percent confidence interval:
##  -0.7699926  3.3065779
## sample estimates:
## mean difference 
##        1.268293
```

```
# bootstrapping
for(i in 1:k) {ci[i, ] <- t.test(FKS_glatt[c(ind[i, ], ind[i, ]+n)]~Task_version[c(ind[i, ], ind[i, ]+n)], data = myData, paired = T, var.equal = T)$conf.int[1:2]}

myCI_FKS_glatt <- quantile(ci[1, ], probs = c(0.05, 0.95))
myCI_FKS_glatt
```

```
##         5%        95% 
## -0.3588061  3.6758793
```

```
ttest_FKS_absorb <- t.test(FKS_absorb ~ Task_version, data = myData, paired = TRUE)
ttest_FKS_absorb
```

```
## 
##  Paired t-test
## 
## data:  FKS_absorb by Task_version
## t = 1.9951, df = 40, p-value = 0.05287
## alternative hypothesis: true mean difference is not equal to 0
## 95 percent confidence interval:
##  -0.01682676  2.60219262
## sample estimates:
## mean difference 
##        1.292683
```

```
ttest_FKS_ges <- t.test(FKS_ges ~ Task_version, data = myData, paired = TRUE)
ttest_FKS_ges
```

```
## 
##  Paired t-test
## 
## data:  FKS_ges by Task_version
## t = 1.7333, df = 40, p-value = 0.09074
## alternative hypothesis: true mean difference is not equal to 0
## 95 percent confidence interval:
##  -0.4251496  5.5471009
## sample estimates:
## mean difference 
##        2.560976
```

```
ttest_FKS_besorg <- t.test(FKS_besorg ~ Task_version, data = myData, paired = TRUE)
ttest_FKS_besorg
```

```
## 
##  Paired t-test
## 
## data:  FKS_besorg by Task_version
## t = -0.55022, df = 40, p-value = 0.5852
## alternative hypothesis: true mean difference is not equal to 0
## 95 percent confidence interval:
##  -1.0258205  0.5867961
## sample estimates:
## mean difference 
##      -0.2195122
```

```
# bootstrapping
for(i in 1:k) {ci[i, ] <- t.test(FKS_besorg[c(ind[i, ], ind[i, ]+n)]~Task_version[c(ind[i, ], ind[i, ]+n)], data = myData, paired = T, var.equal = T)$conf.int[1:2]}

myCI_FKS_besorg <- quantile(ci[1, ], probs = c(0.05, 0.95))
myCI_FKS_besorg
```

```
##         5%        95% 
## -1.3350583  0.0667656
```

```
ttest_FKS_pass <- t.test(FKS_pass ~ Task_version, data = myData, paired = TRUE)
ttest_FKS_pass
```

```
## 
##  Paired t-test
## 
## data:  FKS_pass by Task_version
## t = -0.39746, df = 40, p-value = 0.6931
## alternative hypothesis: true mean difference is not equal to 0
## 95 percent confidence interval:
##  -0.7420638  0.4981613
## sample estimates:
## mean difference 
##      -0.1219512
```

## Analyzing User Experience (UEQ)

Descriptive statistics: Means and SDs.

```
group_by(myData, Task_version) %>%
  summarise(
    count = n(),
    mean_UEQ_Att = mean(UEQ_Att, na.rm = TRUE),
    sd_UEQ_Att = sd(UEQ_Att, na.rm = TRUE)
    )
```

```
## # A tibble: 2 × 4
##   Task_version count mean_UEQ_Att sd_UEQ_Att
##          <int> <int>        <dbl>      <dbl>
## 1            1    41       1.23        0.879
## 2            2    41       0.0773      1.09
```

```
group_by(myData, Task_version) %>%
  summarise(
    count = n(),
    mean_UEQ_Pers = mean(UEQ_Pers, na.rm = TRUE),
    sd_UEQ_Pers = sd(UEQ_Pers, na.rm = TRUE)
    )
```

```
## # A tibble: 2 × 4
##   Task_version count mean_UEQ_Pers sd_UEQ_Pers
##          <int> <int>         <dbl>       <dbl>
## 1            1    41          2.20       0.969
## 2            2    41          2.24       0.947
```

```
group_by(myData, Task_version) %>%
  summarise(
    count = n(),
    mean_UEQ_Eff = mean(UEQ_Eff, na.rm = TRUE),
    sd_UEQ_Eff = sd(UEQ_Eff, na.rm = TRUE)
    )
```

```
## # A tibble: 2 × 4
##   Task_version count mean_UEQ_Eff sd_UEQ_Eff
##          <int> <int>        <dbl>      <dbl>
## 1            1    41        0.811      0.747
## 2            2    41        1.17       0.753
```

```
group_by(myData, Task_version) %>%
  summarise(
    count = n(),
    mean_UEQ_Dep = mean(UEQ_Dep, na.rm = TRUE),
    sd_UEQ_Dep = sd(UEQ_Dep, na.rm = TRUE)
    )
```

```
## # A tibble: 2 × 4
##   Task_version count mean_UEQ_Dep sd_UEQ_Dep
##          <int> <int>        <dbl>      <dbl>
## 1            1    41         1.21      0.800
## 2            2    41         1.07      0.809
```

```
group_by(myData, Task_version) %>%
  summarise(
    count = n(),
    mean_UEQ_Stim = mean(UEQ_Stim, na.rm = TRUE),
    sd_UEQ_Stim = sd(UEQ_Stim, na.rm = TRUE)
    )
```

```
## # A tibble: 2 × 4
##   Task_version count mean_UEQ_Stim sd_UEQ_Stim
##          <int> <int>         <dbl>       <dbl>
## 1            1    41         0.226        1.01
## 2            2    41        -0.530        1.15
```

```
group_by(myData, Task_version) %>%
  summarise(
    count = n(),
    mean_UEQ_Nov = mean(UEQ_Nov, na.rm = TRUE),
    sd_UEQ_Nov = sd(UEQ_Nov, na.rm = TRUE)
    )
```

```
## # A tibble: 2 × 4
##   Task_version count mean_UEQ_Nov sd_UEQ_Nov
##          <int> <int>        <dbl>      <dbl>
## 1            1    41        1          0.851
## 2            2    41       -0.841      1.12
```

Check normal distribution.

```
# compute the difference
d_UEQ_Att <- with(myData, UEQ_Att[Task_version == "1"] - UEQ_Att[Task_version == "2"])
d_UEQ_Pers <- with(myData, UEQ_Pers[Task_version == "1"] - UEQ_Pers[Task_version == "2"])
d_UEQ_Eff <- with(myData, UEQ_Eff[Task_version == "1"] - UEQ_Eff[Task_version == "2"])
d_UEQ_Dep <- with(myData, UEQ_Dep[Task_version == "1"] - UEQ_Dep[Task_version == "2"])
d_UEQ_Stim <- with(myData, UEQ_Stim[Task_version == "1"] - UEQ_Stim[Task_version == "2"])
d_UEQ_Nov <- with(myData, UEQ_Nov[Task_version == "1"] - UEQ_Nov[Task_version == "2"])

# Shapiro-Wilk normality test for the differences
shapiro.test(d_UEQ_Att)
```

```
## 
##  Shapiro-Wilk normality test
## 
## data:  d_UEQ_Att
## W = 0.96315, p-value = 0.2025
```

```
shapiro.test(d_UEQ_Pers)
```

```
## 
##  Shapiro-Wilk normality test
## 
## data:  d_UEQ_Pers
## W = 0.8655, p-value = 0.0001844
```

```
shapiro.test(d_UEQ_Eff)
```

```
## 
##  Shapiro-Wilk normality test
## 
## data:  d_UEQ_Eff
## W = 0.95375, p-value = 0.0948
```

```
shapiro.test(d_UEQ_Dep)
```

```
## 
##  Shapiro-Wilk normality test
## 
## data:  d_UEQ_Dep
## W = 0.95738, p-value = 0.1273
```

```
shapiro.test(d_UEQ_Stim)
```

```
## 
##  Shapiro-Wilk normality test
## 
## data:  d_UEQ_Stim
## W = 0.96997, p-value = 0.3439
```

```
shapiro.test(d_UEQ_Nov)
```

```
## 
##  Shapiro-Wilk normality test
## 
## data:  d_UEQ_Nov
## W = 0.96845, p-value = 0.3067
```

Perform t-tests for data with normal distribution. For data that is
not normally distributed, additional bootstrapping was performed. Effect
sizes were calculated for significant effects.

```
ttest_UEQ_Att <- t.test(UEQ_Att ~ Task_version, data = myData, paired = TRUE)
ttest_UEQ_Att
```

```
## 
##  Paired t-test
## 
## data:  UEQ_Att by Task_version
## t = 5.5326, df = 40, p-value = 2.143e-06
## alternative hypothesis: true mean difference is not equal to 0
## 95 percent confidence interval:
##  0.7327197 1.5761584
## sample estimates:
## mean difference 
##        1.154439
```

```
ttest_UEQ_Pers <- t.test(UEQ_Pers ~ Task_version, data = myData, paired = TRUE)
ttest_UEQ_Pers
```

```
## 
##  Paired t-test
## 
## data:  UEQ_Pers by Task_version
## t = -0.23119, df = 40, p-value = 0.8183
## alternative hypothesis: true mean difference is not equal to 0
## 95 percent confidence interval:
##  -0.3564186  0.2832479
## sample estimates:
## mean difference 
##     -0.03658537
```

```
# bootstrapping
for(i in 1:k) {ci[i, ] <- t.test(UEQ_Pers[c(ind[i, ], ind[i, ]+n)]~Task_version[c(ind[i, ], ind[i, ]+n)], data = myData, paired = T, var.equal = T)$conf.int[1:2]}

myCI_UEQ_Pers <- quantile(ci[1, ], probs = c(0.05, 0.95))
myCI_UEQ_Pers
```

```
##         5%        95% 
## -0.1164732  0.5189122
```

```
ttest_UEQ_Eff <- t.test(UEQ_Eff ~ Task_version, data = myData, paired = TRUE)
ttest_UEQ_Eff
```

```
## 
##  Paired t-test
## 
## data:  UEQ_Eff by Task_version
## t = -2.3555, df = 40, p-value = 0.02349
## alternative hypothesis: true mean difference is not equal to 0
## 95 percent confidence interval:
##  -0.66843783 -0.05107437
## sample estimates:
## mean difference 
##      -0.3597561
```

```
ttest_UEQ_Dep <- t.test(UEQ_Dep ~ Task_version, data = myData, paired = TRUE)
ttest_UEQ_Dep
```

```
## 
##  Paired t-test
## 
## data:  UEQ_Dep by Task_version
## t = 1.0241, df = 40, p-value = 0.3119
## alternative hypothesis: true mean difference is not equal to 0
## 95 percent confidence interval:
##  -0.1305823  0.3988750
## sample estimates:
## mean difference 
##       0.1341463
```

```
ttest_UEQ_Stim <- t.test(UEQ_Stim ~ Task_version, data = myData, paired = TRUE)
ttest_UEQ_Stim
```

```
## 
##  Paired t-test
## 
## data:  UEQ_Stim by Task_version
## t = 3.5731, df = 40, p-value = 0.0009381
## alternative hypothesis: true mean difference is not equal to 0
## 95 percent confidence interval:
##  0.3284214 1.1837737
## sample estimates:
## mean difference 
##       0.7560976
```

```
myData %>% 
cohens_d(UEQ_Stim ~ Task_version, paired = TRUE)
```

```
## # A tibble: 1 × 7
##   .y.      group1 group2 effsize    n1    n2 magnitude
## * <chr>    <chr>  <chr>    <dbl> <int> <int> <ord>    
## 1 UEQ_Stim 1      2        0.558    41    41 moderate
```

```
ttest_UEQ_Nov <- t.test(UEQ_Nov ~ Task_version, data = myData, paired = TRUE)
ttest_UEQ_Nov
```

```
## 
##  Paired t-test
## 
## data:  UEQ_Nov by Task_version
## t = 7.7656, df = 40, p-value = 1.648e-09
## alternative hypothesis: true mean difference is not equal to 0
## 95 percent confidence interval:
##  1.362203 2.320724
## sample estimates:
## mean difference 
##        1.841463
```

```
myData %>% 
cohens_d(UEQ_Nov ~ Task_version, paired = TRUE)
```

```
## # A tibble: 1 × 7
##   .y.     group1 group2 effsize    n1    n2 magnitude
## * <chr>   <chr>  <chr>    <dbl> <int> <int> <ord>    
## 1 UEQ_Nov 1      2         1.21    41    41 large
```

## Analyzing IHR and HRV

Descriptive statistics: Means and SDs.

```
group_by(myData, Task_version) %>%
  summarise(
    count = n(),
    mean_IHR = mean(IHR, na.rm = TRUE),
    sd_IHR = sd(IHR, na.rm = TRUE)
    )
```

```
## # A tibble: 2 × 4
##   Task_version count mean_IHR sd_IHR
##          <int> <int>    <dbl>  <dbl>
## 1            1    41     76.2   10.8
## 2            2    41     76.0   10.6
```

```
group_by(myData, Task_version) %>%
  summarise(
    count = n(),
    mean_HRV = mean(HRV, na.rm = TRUE),
    sd_HRV = sd(HRV, na.rm = TRUE)
    )
```

```
## # A tibble: 2 × 4
##   Task_version count mean_HRV sd_HRV
##          <int> <int>    <dbl>  <dbl>
## 1            1    41     71.9   21.1
## 2            2    41     75.4   24.7
```

Check normal distribution.

```
# compute the difference
d_IHR <- with(myData, IHR[Task_version == "1"] - IHR[Task_version == "2"])
d_HRV <- with(myData, HRV[Task_version == "1"] - HRV[Task_version == "2"])

# Shapiro-Wilk normality test for the differences
shapiro.test(d_IHR)
```

```
## 
##  Shapiro-Wilk normality test
## 
## data:  d_IHR
## W = 0.97643, p-value = 0.5913
```

```
shapiro.test(d_HRV)
```

```
## 
##  Shapiro-Wilk normality test
## 
## data:  d_HRV
## W = 0.88779, p-value = 0.001173
```

Perform t-tests for data with normal distribution. For data that is
not normally distributed, additional bootstrapping was performed. Effect
sizes were calculated for significant effects.

```
ttest_IHR <- t.test(IHR ~ Task_version, data = myData, paired = TRUE)
ttest_IHR
```

```
## 
##  Paired t-test
## 
## data:  IHR by Task_version
## t = 0.27803, df = 37, p-value = 0.7825
## alternative hypothesis: true mean difference is not equal to 0
## 95 percent confidence interval:
##  -1.012829  1.334987
## sample estimates:
## mean difference 
##       0.1610788
```

```
ttest_HRV <- t.test(HRV ~ Task_version, data = myData, paired = TRUE)
ttest_HRV
```

```
## 
##  Paired t-test
## 
## data:  HRV by Task_version
## t = -0.95235, df = 37, p-value = 0.3471
## alternative hypothesis: true mean difference is not equal to 0
## 95 percent confidence interval:
##  -10.708697   3.860759
## sample estimates:
## mean difference 
##       -3.423969
```

```
# bootstrapping
for(i in 1:k) {ci[i, ] <- t.test(HRV[c(ind[i, ], ind[i, ]+n)]~Task_version[c(ind[i, ], ind[i, ]+n)], data = myData, paired = T, var.equal = T)$conf.int[1:2]}

myCI_HRV <- quantile(ci[1, ], probs = c(0.05, 0.95))
myCI_HRV
```

```
##         5%        95% 
## -10.009259   3.645257
```
